# Computational Modelling of Novelty Detection in the Mismatch Negativity Protocols and Its Impairments in Schizophrenia

**DOI:** 10.1111/ejn.70453

**Published:** 2026-03-20

**Authors:** Ahmed Eissa, Jan Fredrik Kismul, Atle Bråthen Pentz, Torbjørn Elvsåshagen, Christoph Metzner, Ibrahim Akkouh, Srdjan Djurovic, Alexey Shadrin, Marja‐Leena Linne, Gaute T. Einevoll, Ole A. Andreassen, Tuomo Mäki‐Marttunen

**Affiliations:** ^1^ Faculty of Medicine and Health Technology Tampere University Tampere Finland; ^2^ Division of Mental Health and Addiction Oslo University Hospital Oslo Norway; ^3^ Institute of Clinical Medicine University of Oslo Oslo Norway; ^4^ Institute of Basic Medical Sciences University of Oslo Oslo Norway; ^5^ Department of Child and Adolescent Psychiatry Charité Universitätsmedizin Berlin Berlin Germany; ^6^ School of Physics, Engineering and Computer Science University of Hertfordshire Hatfield UK; ^7^ Department of Medical Genetics Oslo University Hospital Oslo Norway; ^8^ Department of Physics Norwegian University of Life Sciences Ås Norway; ^9^ Department of Physics University of Oslo Oslo Norway; ^10^ Department of Biosciences University of Oslo Oslo Norway

**Keywords:** deviance detection, gene expression, MMN, schizophrenia and genetics, spiking neuronal network

## Abstract

The human auditory system rapidly distinguishes between novel and familiar sounds, a process reflected in mismatch negativity (MMN), an electroencephalogram (EEG)‐based biomarker of auditory novelty detection. MMN is impaired in psychiatric conditions, most notably schizophrenia (SCZ), yet the neuronal mechanisms underlying this deficit remain unclear. Here, we combined computational modelling and genetic analyses to investigate how SCZ‐associated cellular abnormalities affect auditory novelty detection. We developed an integrate‐and‐fire spiking network model capable of detecting four types of auditory novelty, including stimulus omission. Based on assumptions of short‐term depressing synapses between the subpopulations of the network and the existence of neuronal inputs that are phase‐locked to the rhythm of the recently experienced stimulus sequence, we showed that the model reliably reproduced MMN‐like novelty detection and allowed systematic testing of SCZ‐related cellular alterations. We also demonstrated that the required phase locking can theoretically be achieved in a synfire chain network exhibiting spike‐timing dependent plasticity (STDP) in its feedback synapses that becomes entrained to the rhythmic stimulus. Simulations of our novelty‐detecting network revealed that both reduced pyramidal cell excitability, linked to ion channel dysfunction, and decreased spine density impaired novelty detection, with the latter producing stronger deficits. Our work provides a flexible spiking network model of auditory novelty detection that can link cellular‐level abnormalities to measurable MMN deficits, improving their mechanistic interpretation and helping to explain the heterogeneity of SCZ.

## Graphical abstract

The mechanisms of novelty detection in the mismatch negativity (MMN) protocol and its disturbances in schizophrenia (SCZ) remain unclear. Here, we developed an integrate‐and‐fire (IAF) spiking neuronal network model to detect multiple types of auditory novelty. For this, we assumed the existence of excitatory (ES) and inhibitory (IS) stimulus‐encoding populations, an excitatory population that receives inputs phase‐locked to the stimulus rate (EP), and an output population (EO) that receives synaptic contacts exhibiting short‐term depression from the excitatory populations (left). With additional populations for alternative stimulus frequencies and durations, we showed that the network is able to detect four types of deviants: frequency deviants, stimulus omissions, duration deviants where the stimulus duration was longer for a deviant, and inverse duration deviants where it was shorter (upper right). Combining the model with postmortem genetics data on ion‐channel expression in SCZ and control subjects and a biophysically detailed single‐cell model that helped to convert these gene‐expression differences to a parameter change in the IAF model, we showed that the model predicted an impaired novelty‐detection response in most MMN protocols for SCZ (lower left). A generic decrease in spine density had a similar effect (lower right). Although yet a theoretical model, our framework provides an example of a network capable of multiple types of novelty detection with neurobiologically plausible mechanisms that can be used to explore the cellular and network level phenotypes in psychiatric disorders and their effects on MMN.

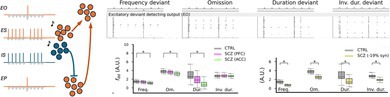

**Abbreviations**
AMPAR: α‐amino‐3‐hydroxy‐5‐methyl‐4‐isoxazolepropionic acid receptor
ACC: Anterior cingulate cortex
AUC: Area under curve EEG Electroencephalogram
EEG: Electroencephalogram
ERP: Event‐related potential
GABAR: γ‐aminobutyric acid
IAF: Integrate‐and‐fire
MMN: Mismatch negativity
NMDAR: N‐methyl‐D‐aspartate receptor
PFC: Prefrontal cortex
SCZ: Schizophrenia
STDP: Spike‐timing dependent plasticity
SSA: Stimulus‐specific adaptation Population abbreviations
ES: Excitatory, stimulus‐encoding
IS: Inhibitory, stimulus‐encoding
ED: Excitatory, encodes frequency‐deviant stimulus
ID: Inhibitory, encodes frequency‐deviant stimulus
ESD: Excitatory, stimulus‐encoding, delayed‐activating
ISD: Inhibitory, stimulus‐encoding, delayed‐activating
EDD: Excitatory, encodes frequency‐deviant stimulus, delayed‐activating
IDD: Inhibitory, encodes frequency‐deviant stimulus, delayed‐activating
EP: Excitatory, receives phase‐locked inputs
EP2: Excitatory, receives phase‐locked inputs with alternative phase
EO: Excitatory output population
CO: Cortical output population

## 1 Introduction

The human auditory system is highly effective in distinguishing between novel and familiar sounds and auditory patterns. Detecting novel auditory stimulus can help us shift attention and plan our actions accordingly, but it also generates event‐related potentials (ERPs) that can be measured using an electroencephalogram (EEG) in both conscious and unconscious modes of operation (Alho 1992). Mismatch negativity (MMN), defined as the difference between ERPs elicited by novel and familiar tones, is the most widely used EEG biomarker of auditory novelty detection (Näätänen 1982; Lakatos et al. 2020). MMN is clinically relevant because it is impaired in psychiatric disorders, particularly in schizophrenia (SCZ) (Light and Näätänen 2013). However, due to the complexity of the auditory system, the neuronal mechanisms of auditory novelty detection in general, as well as its impairment in SCZ, remain incompletely understood. Elucidating these mechanisms may help bridge cellular‐level alterations associated with SCZ to clinically observed deficits.

Computational modelling provides an efficient way of studying novelty detection and integrating disease‐associated information across different modalities (such as genetic data and cellular‐level structural data) to test hypotheses on how novelty detection is affected in brain disorders. Two mechanistic accounts have dominated explanations of MMN (May and Tiitinen 2010). Stimulus‐specific adaptation (SSA) proposes that neurons tuned to stimulus features (e.g., frequency or duration) adapt to repeated presentation of the standard tone, so that a physically deviant tone elicits a larger response and thus an MMN signal. The model‐adjustment (predictive‐coding) hypothesis instead proposes that the brain builds an internal model of recent stimuli and that a mismatch between model‐based predictions and incoming input produces the MMN (Garrido et al. 2009; Carbajal and Malmierca 2018). In computational work, researchers typically follow one of two routes: biophysically detailed models that implement SSA in spiking networks (Han et al. 2023) or higher level predictive‐coding/model‐adjustment formulations that operate at an abstract, nonspiking level (Lieder et al. 2013; Adams et al. 2022; Poublan‐Couzardot et al. 2023). Hybrid approaches have also been proposed that combine elements of both (Wacongne et al. 2012; López‐Caballero et al. 2025). However, certain MMN phenomena—most notably omission MMN—are difficult to account for with a physiologically realistic spiking neuronal network model since they seem to require mechanisms that generate expectation‐related activity in the absence of sensory input (May and Tiitinen 2010; Garrido et al. 2009; Carbajal and Malmierca 2018). Motivated by proposals that oscillatory activity in neurons could help to instantiate such expectation signals (May and Tiitinen 2010), we developed a biophysically plausible spiking network that combines SSA‐like adaptation with inputs phase‐locked to the stimulus rhythm. Although there is no experimental evidence of such an architecture, we show here that the phase locking to a rhythmic simulus can be formed in a synfire chain with weak, Hebbian‐like plasticity‐obeying feedback synapses. Our suggested novelty detection network architecture allows the model to produce novelty responses to a range of deviant types—including omissions—while keeping a clear link to cellular‐level mechanisms that may be altered in psychiatric disease.

In this work, we employed computational modelling of spiking neuronal networks to investigate the auditory novelty detection and the ways in which it is compromised by SCZ‐associated cellular‐level abnormalities. We developed an integrate‐and‐fire (IAF) model network capable of detecting four types of auditory novelty frequently used in the MMN paradigm: a frequency deviant, an omitted stimulus, a deviant with a longer duration than the standard tone (henceforth the “duration deviant”), and, finally, a deviant with a shorter duration than the standard tone (henceforth the “inverse duration deviant”). We demonstrated the robustness of our proposed network model in novelty detection under a variety of conditions and examined how SCZ‐associated reductions in pyramidal cell excitability, suggested by alterations in ion channel expression (Hoffman et al. 2019; Mäki‐Marttunen et al. 2024), and a decrease in spine density (Shelton et al. 2015) affect novelty detection. Although both alterations impaired deviance detection, our simulations suggested that decreased spine density exerts stronger effects than decreased neuronal excitability. This work therefore serves a dual purpose: to introduce a mechanistic spiking network framework for auditory novelty detection and to apply it in the context of SCZ to identify how cellular abnormalities may translate into measurable MMN deficits. By linking cellular‐level alterations to measurable impairments in auditory novelty detection, our model provides a framework for developing targeted therapeutic strategies and stratifying patients based on distinct neurophysiological profiles.

## 2 Methods

### 2.1 Network Model for Novelty Detection in the Auditory Pathway

The MMN signal can be registered using many different protocols, where the deviant sound is different from the standard sound in terms of pitch, phoneme (if human voice‐based stimuli used), duration, interstimulus interval, or intensity, or even when the stimulus is omitted (Garrido et al. 2009). The aim of this work was to present a spiking neuronal network model capable of detecting mismatches of four types, namely, a frequency deviant, an omitted stimulus, a duration deviant, and an inverse duration deviant. To describe the neuronal mechanisms that permit novelty detection, we introduced a spiking neuronal network of multiple neuron populations.

We made the following assumptions (their validity is discussed in Section 4):

1. There are excitatory and inhibitory populations of neurons that selectively respond to each tone, and the excitatory neuron populations project to other populations by short‐term depressing synapses.
2. Some of these populations respond upon receiving either short or long‐lived auditory stimulus, while some populations require a long‐lived auditory stimulus to be activated.
3. There are populations of neurons (separate from the above) that are phase‐locked to a 2‐Hz stimulus presentation frequency with different phase shifts.
4. These neuron populations interact with an “output” population that detects auditory mismatches of different type.

**FIGURE 1:**
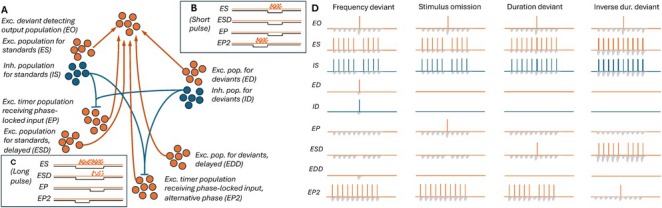
Illustration of the model network and the hypothesized mechanisms of deviance detection. See Figures S1–S4 for a breakdown of the hypothesized mechanisms.

To develop a network model to meet this aim under the above assumptions, we considered excitatory (ES) and inhibitory (IS) populations that selectively responded to the standard (S) tone, and excitatory (ED) and inhibitory (ID) populations that selectively responded to the deviant (D) tone. We also modelled two delayed‐activating populations excitatory, stimulus‐encoding, delayed‐activating (ESD), excitatory, encodes frequency‐deviant stimulus, delayed‐activating (EDD) that were similar to ES and ED but required longer inputs to fire. To activate these neurons, we used a stimulus that lasted twice as long as the standard tone and ended at the same time. Furthermore, we modelled an excitatory neuron population that received inputs from a population that was phase‐locked to a 2‐Hz stimulus presentation rate with a phase that was coincident with standard stimulus (EP), as well as another population (EP2) with a phase aligned with the first half of the longer duration deviant. Finally, we modelled an excitatory population that received inputs from all six excitatory populations (unidirectional synapses with a connection probability 0.5) and functions as an output population (EO). All excitatory synaptic connections were assumed to be short‐term depressing, which allowed the repeated standards to induce a different network response than the deviants. The inhibitory connections (from neurons in IS and ID to neurons in EP and EP2) were likewise formed with a connection probability 0.5 but exhibited no short‐term depression. The network is illustrated in Figure 1A; step‐by‐step schematics are shown in Figures S1–S4.

We here modelled only one additional specific frequency‐tuned population in addition to the standard tone‐tuned population and a pacemaking population tuned to particular phases of a particular stimulus presentation rate (2 Hz), but our framework can be extended to describe any number of such deviants and pacemaking signals. In this work, the short stimuli lasted 50 ms, and the long stimuli lasted for 100 ms such that they started 50 ms before the onset of the short stimuli.

We hypothesized that the network described above could perform the deviance detection by the following mechanisms. First, the ES and ED populations project to the output EO population via depressing synapses. Repeated standards elicit weaker responses than deviants, such that EO fires to a deviant but not to repeated standards (Figure S1). Second, the omission detecting population EP receives excitatory inputs from phase‐locked neurons (not explicitly modelled) that were assumed to be independent of the auditory stimuli (however, the activation of these neurons may be dependent on the history of the auditory stimuli through plasticity mechanisms). These inputs are counteracted by inhibitory inputs from the frequency‐sensitive neuron populations IS and ID, and thus, the EP population only fires when none of the frequency‐sensitive neuron populations are active (Figure 1B). When the omission detecting population EP fires, it activates the output population (Figure S2). Third, the delayed‐activating population ESD fires in response to a long but not short stimulus (Figure 1B,C). At the introduction of the first longer stimulus among shorter standards, the ESD fires and makes the output population fire as well (Figure S3). Fourth, the long stimulus inhibits not only the EP but also the EP2 population (Figure 1C) that would otherwise fire at the phase preceding the phase of the EP population (Figure 1B). Similar to EP, the EP2 population projects to the output population with short‐term depressing synapses. EP2 thus remains silent in response to recurring long standards but fires when a shorter lived stimulus is used as a deviant (Figure 1C) and causes the output population to fire (Figure S4). These mechanisms are illustrated in Figure 1D—see Section 3 for our approach for fitting the model to reproduce these mechanisms.

We modelled the neurons as leaky IAF units that received conductance‐based synaptic inputs mediated by α‐amino‐3‐hydroxy‐5‐methyl‐4‐isoxazolepropionic acid receptors (AMPARs), N‐methyl‐D‐aspartate receptors (NMDARs), and γ‐aminobutyric acid (GABARs) in addition to square‐pulse currents that were timed to correspond to inputs from stimulus‐frequency‐coding and pacemaker neurons. Each population consisted of N=40 neurons, and we used the single‐compartment model with the same membrane properties for both inhibitory and excitatory neurons. The glutamatergic inputs from the ES, ED, EP, ESD, EDD, and EP2 populations to the output population EO were short‐term depressing according to the following scheme (adapted from Wang 1999):

(1) $$\frac{dD}{dt}=-p_{v}D\underset{j}{\sum }\delta (t-t_{j}^{-})+(1-D)/\tau_{D},$$

where D is the fraction of releasable vesicles, $p_{v}$ is the release fraction per spike, and $\tau_{D}$ is the time constant of recovery from depression. The term $t_{j}^{-}$ represents the time instants of the presynaptic spikes.

### 2.2 Optimization of the Model Parameters

We used the grid search method to achieve model parameters that yielded a good deviance detection performance. We ran the four MMN experiments (the frequency deviant, the omission of stimulus, the duration deviant, and the inverse duration deviant) for a large number of parameter sets (5 stimulus amplitudes × 5 exc. synaptic conductances from the ES and ED populations × 5 exc. synaptic conductances from the EP and EP2 populations × 7 exc. synaptic conductances from the ESD and EDD populations × 2 NMDA/AMPA ratios × 6 inh. synaptic conductances × 2 depression strengths × 2 membrane capacitances for the ESD and EDD populations; 42,000 parameter sets in total). The tested parameter ranges are shown in Table S2A. We considered networks where ≥80% of the output (EO) neurons fired once or more for the deviant, and the number of EO spikes following the deviant was at least six times an average number of EO spikes following a standard as acceptable models.

### 2.3 Quantification of the Deviance Detection

To assess the network activity, we quantified deviance detection using an index inspired by the MMN, calculating the average numbers of spikes induced by the deviant stimulus (or omission of stimulus) ($N_{deviant}$) and that induced by the standard ($N_{standard}$) in the output population. For each stimulus, we included the spikes elicited from 150 ms prior to the expected onset of the stimulus to 350 ms after the onset of the stimulus and obtained the firing rates $f_{deviant}$ and $f_{standard}$ by normalizing the numbers of spikes by the interval (0.5 s). We then defined the deviance detection index as

(2) $$f_{\text{dd}}=f_{\text{deviant}}-f_{\text{standard}}.$$

### 2.4 Modelling of Spike‐Timing‐Dependent Plasticity (STDP)

In Section 3.3, we tested the entrainment of a network organized as a synfire chain to external auditory stimuli arriving at a certain rhythm via STDP. We implemented the base neuron population (N=50 neurons) and 70 (unless otherwise stated) synfire chain populations (N=50 neurons in each population) as IAF neurons receiving (static) AMPAR‐mediated synaptic currents that ensured a progression of the signal through the chain. We introduced a 5‐ms synaptic transmission delay in these feedforward synapses. To avoid runaway excitation, we introduced inhibitory populations, one for each excitatory population, that were bidirectionally connected to their corresponding excitatory populations.

From each excitatory population within the chain, we introduced STDP‐exhibiting feedback synapses back to the base population. We implemented the synapse weight update rules using the standard STDP formalism available in Brian 2 (Stimberg et al. 2019) as follows:

(3) $$\frac{dA_{pre}}{dt}=-\frac{A_{pre}}{\tau_{A}}+A^{+}\underset{k}{\sum }\delta (t-t_{pre}^{\left(k\right)}),$$

(4) $$\frac{dA_{post}}{dt}=-\frac{A_{post}}{\tau_{A}}+A^{-}\underset{k}{\sum }\delta (t-t_{post}^{\left(k\right)}).$$

Here, $A_{pre}>0$ and $A_{post}<0$ are dynamic variables representing presynaptic and postsynaptic traces, respectively; $A^{+}>0,\;A^{-}<0$ are the jump sizes, $\tau_{A}=20$ is the decay constant of the traces, and δ(·) denotes the Dirac delta. When a presynaptic spike occurred, an AMPAR‐mediated current was initiated in the postsynaptic neuron using the current synaptic weight w; the *pre*synaptic trace was increased by the constant $A^{+}>0$ (see Equation (3)), and the synaptic weight was decreased by the current value of the *post*synaptic trace:

(5) $$w\leftarrow w+A_{post}\;\text{at}\;t=t_{pre}.$$

When a postsynaptic spike occurred, the *post*synaptic trace was decreased by the constant $A^{-}$ (Equation (4)) and the synaptic weight was increased by the current value of the *pre*synaptic trace:

(6) $$w\leftarrow w+A_{pre}\;\text{at}\;t=t_{post}.$$

This meant that if both presynaptic and postsynaptic neurons fired, a pre→post order caused the presynaptic trace to be increased (Equation (3)) before the synapse weight w was increased by its value (Equation (6)) during the postsynaptic spike, leading to a strengthening of the synapse, while a post→pre order caused the postsynaptic trace to become more negative (Equation (4)) before its value was added to w (Equation (5)), leading to a weakening of the synapse. Weight updates were clipped to remain within fixed bounds (0 to 2400 pS). This implementation allowed synaptic efficacy to evolve according to the relative timing of presynaptic and postsynaptic activities, following a standard pair‐based STDP rule where small interspike intervals had the largest effects on the synapse weight w. The learning rate parameters were set asymmetrically, and the values of $A^{+}$ and $A^{-}$ were optimized in a grid search (see Section 3). The initial synaptic weights were set to a low value (the value of $A^{+}$ was used), which did not affect the signal propagation through the synfire chain.

To analyze the spiking activity and the entrainment, we quantified the synchronized population activity using the burst detection method that divides the spiking activity to separate bursts if the interval between two consecutive spikes exceeds a constant (Chiappalone et al. 2006; Mäki‐Marttunen et al. 2013).—here, we used a maximal interspike interval of 50 ms. For each simulation, we counted the poststimulus cycles of activity (population bursts) that were appropriately timed compared to the last cycle, namely, that the first spike in the population burst occurred no earlier than 450 ms after the last spike in the previous burst and the last spike in the burst occurred no later than 550 ms after the last spike in the previous burst. We also made sure that there were no exceedingly long cycles of activity, namely, population bursts that lasted for more than 75 ms.

### 2.5 Use of Genetic Data in the Modelling

To study the effects of altered expression of ion channels on deviance detection, we used the CommonMind RNA expression data (Hoffman et al. 2019) from two regions: the anterior cingulate cortex (ACC, N=478 of which 251 were controls and 227 SCZ patients) and prefrontal cortex (PFC, N=426 of which 215 were controls and 211 SCZ patients). We followed the approach of Mäki‐Marttunen et al. (2024) in applying these data for modelling. Unlike the study of Mäki‐Marttunen et al. (2024) that employed biochemically and biophysically detailed neuron modelling, here, we used simplified IAF models that do not permit information on ion channel expression to be integrated into the model. We thus used the Hay model (Hay et al. 2011) as a proxy to estimate the effects of altered ion channel expression on pyramidal cell firing behavior.

In short, we first used a list of all SCZ risk genes associated with fast or slow neurotransmission (Devor et al. 2017) and the sister genes in their gene families (Table S2 in Mäki‐Marttunen et al. 2024). We then extracted the high‐risk variants within these genes from (Trubetskoy et al. 2022). While the study of Trubetskoy et al. (2022) applied several complementary strategies to assign single‐nucleotide polymorphisms (SNPs) to credible genes, in our analysis, we used a simpler positional approach: SNPs were assigned to genes if their coordinates fell within gene boundaries. Genes were retained as genes of interest if at least one SNP within their boundaries had a p‐value smaller than $5\cdot 10^{-6}$. In particular, we focused on the genes whose effects we could model using the model of Hay et al. (2011). (Table S1A). We complemented this set of genes by sets of genes that were differentially expressed in SCZ compared to healthy controls (CTRL) (Table S1B). To do this, we analyzed the CommonMind data of postmortem RNA expression in PFC and ACC. The expression data were normalized using the DESeq2 method (Love et al. 2014). We also employed an imputation tool, CIBERSORTx (Steen et al. 2020), to obtain estimates of neuronal expression in these brain regions instead of the bulk expression using a reference single‐cell RNA dataset (Zhang et al. 2016). We only considered the genes that were successfully imputed, and furthermore, we restricted our analysis on genes that showed a “medium” or “high” level of protein expression in neuronal cells of cerebral cortex or had a high specificity for expression in excitatory neurons according to the single‐cell RNA sequencing data of Human Protein Atlas. We then normalized each gene expression of each subject by the average expression of the underlying gene in the 251 (ACC) or 215 (PFC) controls. When there were multiple SCZ hit genes affecting a single model parameter, we used the average of the expression‐level factors to obtain a single factor.

The pyramidal cell model of Hay et al. (2011) consisted of 196 compartments and described the following ionic currents: high‐voltage activated Ca^2+^ current ($I_{\mathrm{Ca},\mathrm{HVA}}$), low‐voltage activated (LVA) Ca^2+^ current ($I_{\mathrm{Ca},\mathrm{LVA}}$), nonspecific cationic current ($I_{h}$), transient ($I_{\mathrm{Na},t}$) and persistent ($I_{\mathrm{Na},p}$) Na^+^ currents, muscarinic K^+^ current ($I_{m}$), transient ($I_{K,t}$) and persistent ($I_{K,p}$) K^+^ currents, Ca^2+^‐activated SK current ($I_{\text{SK}}$), Kv3.1‐mediated K^+^ current $I_{\text{Kv}3.1}$, and the leak current ($I_{\text{leak}}$). Changes in the expression of the subunits contributing to these currents were modelled by altered maximal conductance ($\bar{g}$) of the underlying current. Similar to (Mäki‐Marttunen et al. 2024), we attributed the expression of CACNA1C and CACNA1D to $I_{\mathrm{Ca},\mathrm{HVA}}$, that of CACNA1I to $I_{\mathrm{Ca},\mathrm{LVA}}$, that of HCN1 to $I_{h}$, and that of KCNQ3 to $I_{m}$, those of KCNB1 and KCND3 to $I_{K,p}$, and that of SCN1B to $I_{\mathrm{Na},t}$.

To determine the effects of altered expression on intrinsic excitability, we simulated the pyramidal cell spiking response to a set of somatic currents (0 to 1.0 nA in intervals of 0.1 nA). We simulated the model separately for each subject, where the conductances were adapted in a subject‐ and current species‐specific manner as explained above. We determined the f‐I curves, that is, the spiking frequencies during the last 15.5 s of the current injection with respect to the current amplitude. The obtained results, and in particular, the obtained subject‐wise areas under curve (AUC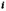), were then refined for further use in IAF simulations in one of the two ways:

1. The membrane capacitance of the IAF model for subject i was multiplied by $\frac{\bar{\text{AUC}}}{{\text{AUC}}_{i}}2$, where $\bar{\text{AUC}}$ is the average AUC across control subjects.
2. The membrane capacitance of the cortex‐adapted IAF model for subject i was fitted to yield the exactly same AUC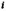 in a corresponding f‐I experiment with the IAF model.

We then averaged across the membrane capacitances of the SCZ population to yield an estimate of how much the SCZ diagnosis, mediated by altered ion channel expression, affects the membrane capacitance.

### 2.6 Statistical Tests

We used the Wilcoxon rank‐sum test (U‐test) for group differences between SCZ and CTRL subjects (nonparametric; two‐sided; normal approximation). We corrected for multiple (4, i.e., the number of protocols) comparisons using the Bonferroni correction.

### 2.7 Code Availability

All simulations were run using Brian 2 (Stimberg et al. 2019) or NEURON simulator (Hines and Carnevale 2001) v. 8.2.6, using Python (3.9.20) interface. Our simulation scripts are available at ModelDB, accession number 2019882 (https://modeldb.science/2019882).

## 3 Results

### 3.1 Development of a Spiking Network Model Capable of Novelty Detection in the Frequency and Omission‐Based MMN

**FIGURE 2:**
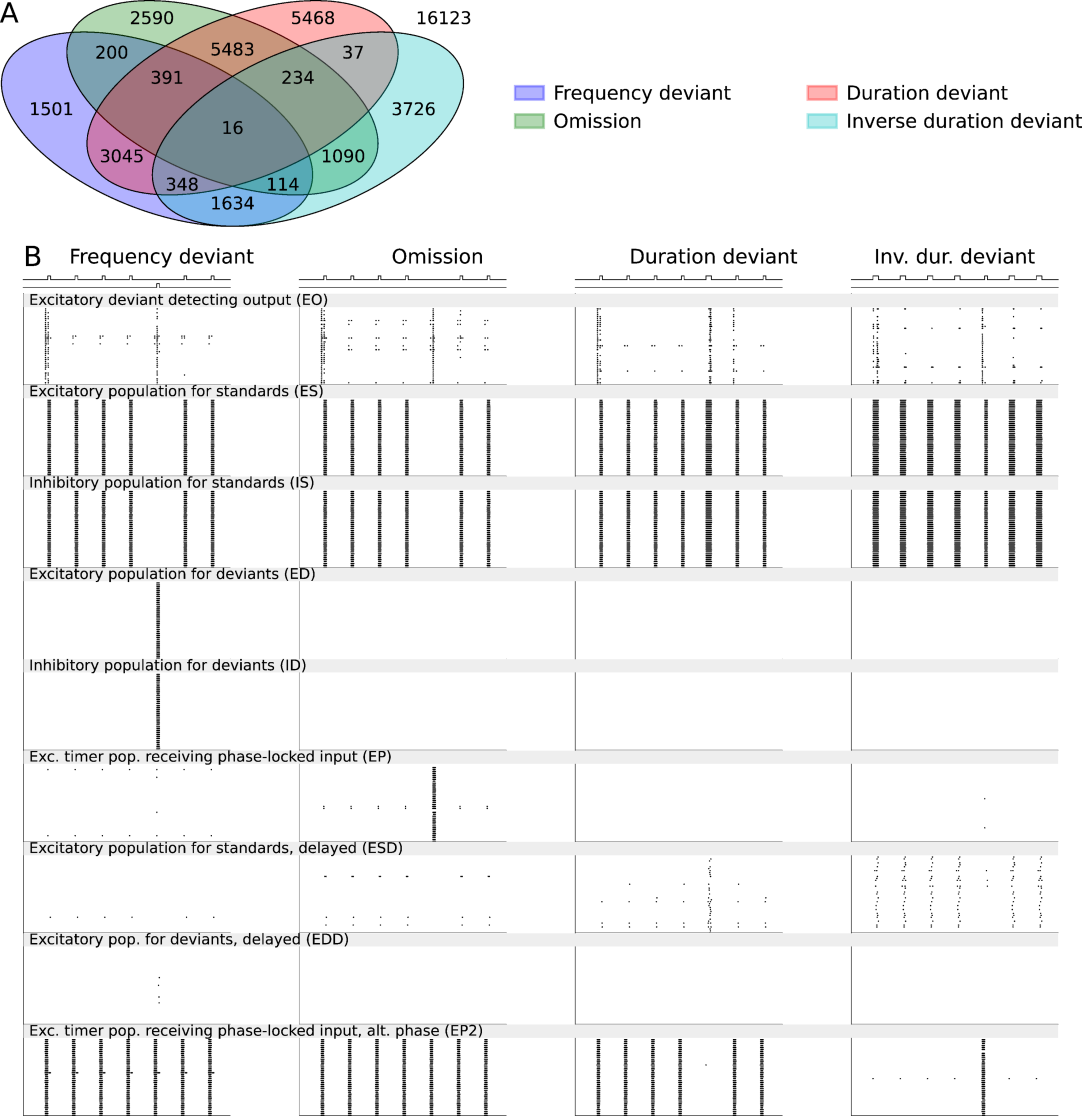
Fitting of the spiking network model for novelty detection in four different types of MMN protocols and the firing behavior of the subpopulations in the four protocols. (A) The Venn diagram showing the number of parameter sets (out of 42,000) that yielded an acceptable novelty detection in the frequency deviant (blue), omission (green), duration deviant (red), and inverse duration deviant (cyan) MMN protocols and their intersection. Sixteen parameter sets yielded an acceptable novelty detection in all four protocols. (B) Population spike raster plots of the nine subpopulations (40 neurons in each) in the four MMN protocols according to one of the 16 acceptable models.

To describe the novelty detection based on spiking neuronal network dynamics in health and disease, we introduced a network of multiple neuron populations (see Section 2 and Figure 1). We first ran a series of grid search experiments to find an approximate range of parameters (stimulus amplitudes, synaptic conductances, membrane capacitances, and the depression strength) within which appropriate spiking and a different response for deviants versus standards were achieved. We then ran a large grid search to optimize the novelty detection (see Table S2A). We achieved strong deviance detection for 7249, 10,118, 15,022, and 7199 parameter sets (out of 42,000) in the frequency‐deviant, stimulus‐omission, duration deviant, and the inverse duration deviant protocols, respectively (Figure 2A). A total of 16 parameter sets supported detection across all four protocols (Figure 2A)—these parameters are listed in Table S2B. The behavior of all nine neuron populations in response to the four MMN protocols is illustrated in Figure 2B.

Taken together, our grid‐search method yielded spiking networks that effectively detected novelty in typical MMN stimulus protocols.

### 3.2 The Novelty‐Detecting Network Functions Robustly

**FIGURE 3:**
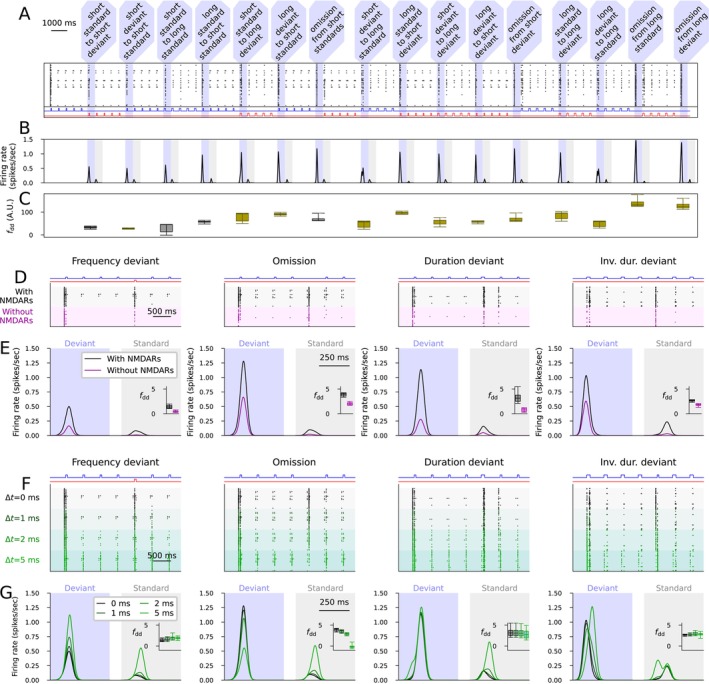
The novelty‐detecting network functions well in the roving paradigm, shows NMDAR‐dependency, and is robust against introduction of synaptic delays. (A–C) Experiments with an alternative stimulation sequence where the four stimuli (short standard, short deviant, long standard, long deviant) are repeated in sequences of five tones and all possible transitions between them and all omissions are included. (A) The output population activity according to the model with the default parameter set. The shaded areas represent the deviants, that is, transitions to a new type of tone or omissions. The insets below the graph show the stimuli corresponding to the standard (blue) and deviant (red) tones across time. (B) The experiment of (A) was repeated for all 16 models. The plots show the average firing rate curves induced by the deviants (blue shading) and those induced by the previous tone (gray shading). (C) Box plots of the deviance detection index determined from the data of (B). The magenta bars represent the MMN protocols used in Figure 2, and the cyan bars represent the alternative deviants not used for fitting the model. (D, E) The deviance detection with and without NMDAR‐mediated currents. (D) The output population activity according to the model with the default parameter set in the presence (black) and absence (magenta) of NMDAR‐mediated currents. (E) Average (across the 16 models and 10 repetitions with different random number seeds) firing rate curves induced by the deviants (blue shading) and those induced by the previous tone (gray shading) in the four MMN protocols in the presence (black) and absence (magenta) of NMDAR‐mediated currents. The firing rate curve was obtained by convolving the spike trains with a Gaussian with a 25‐ms SD. The insets show the box plots of the deviance detection indices in each protocol. (F, G) The deviance detection with and without synaptic delays. (F) The output population activity according to the model with the default parameter set in the absence (black) and presence of 1 (dark green), 2 (green) or 5 (light green) ms synaptic delays. (G) Average (across the 16 models and 10 repetitions with different random number seeds) firing rate curves in the four MMN protocols in the absence (black) and presence (green) of synaptic delays. The insets show the box plots of the deviance detection indices in each protocol.

To show the robustness of the novelty‐detecting network, we validated the model by additional experiments. First, we showed that the network can detect novelty in a roving deviant paradigm where a sequence of five identical stimuli (whether short or long tones of the standard or deviant frequency) forms a new standard after each transition (Figure 3A,B). In the same experiment, we also tested the omission of stimulus when each of the four types of tones were used as a standard (Figure 3A,B). The deviance detection indices were of similar magnitude or larger in these transitions compared to the MMN protocols used in Figure 2 (Figure 3C).

We also simulated the deviance detection in all four MMN protocols when the NMDARs were blocked. Similar to experimental observations (Farley et al. 2010), the simulations with NMDAR blockage showed a decreased response to both deviants and standards (Figure 3D,E). Our simulations, however, suggested that the deviance detection index was also decreased in the absence of NMDAR activation (Figure 3E).

Biological networks include delays consisting of axonal and dendritic signal conduction and a presynaptic neurotransmitter release before a presynaptic AP induces AMPAR, NMDAR, or GABAR‐mediated currents in the postsynaptic neuron, which is not taken into account in the model. We tested the functionality of the novelty detection network in the presence of neurotransmission delays. The deviance detection remains unchanged in all MMN protocols when small delays (Δt=1 or 2 ms) were used, but the detection of stimulus omission was lost when a large delay (Δt=5 ms) was used (Figure 3F,G).

We next tested the contribution of individual populations in the deviance detection. In these experiments, we removed some of the populations and performed the four different experiments without the selected populations and recorded the output population firing activity. When the EP2 population that was responsive during the alternative phase of the 2‐Hz stimulus presentation was removed, the detection of inverse duration deviants was lost, while the other three types of deviants were detected (Figure S5A). Removing both EP and EP2 abolished omission and inverse duration detection but preserved frequency and duration detection (Figure S5B). By contrast, removing the delayed‐activating populations (ESD, EDD) alongside the EP2 population abolished the detection of all duration deviants but preserved the detection of frequency deviants and omitted stimuli (Figure S5C). When only the tone‐sensitive populations (ES, ED, IS, and ID) and the output population were in place while others were removed, the network only detected the frequency deviants (Figure S5D).

We verified that the network was robust against small changes in network size and variability in neuronal excitability (Figure S6A,B). The network detected deviants when its size was halved (20 neurons) or increased to 60 neurons (Figure S6A) and when the variability of the membrane capacitance of the neurons was reduced to 0.2× mean($C_{m}$) or increased to 0.4× mean($C_{m}$) (Figure S6B). However, given too little variability in the membrane capacitance of the neurons (SD(τ) ≤ 0.1× mean(τ)), the inverse duration deviants were no more detected (Figure S6B). We also made sure the network does not produce a deviance detection artifact due to the phase‐locked neuron activity when random auditory stimuli were given as inputs instead of the ryhthmic tone inputs (Figure S6C).

While the activation of the stimulus‐encoding neuron populations (ES, ED) can be expected to be timely, the phase‐locked populations may show large deviation in the timing of its activity. Here, we show that the detection of omitted stimuli is not dependent on a perfectly timed activation of the 2‐Hz phase‐locked populations. To do this, we randomized a phase for each of the phase‐locked neuron from a uniform distribution [$t_{0}-\theta ,t_{0}+\theta$] where the jitter magnitude term θ ranged from 0 (no jitter) to 20 ms. A jitter of ±2 to 5 ms had little effect on the deviance detection in any of the MMN protocols, but a jitter of ±20 ms almost abolished the detection of omissions and also mildly impaired the detection of inverse duration deviants (Figure S6D,E). Finally, we tested that the network also detected a double deviant (deviant in both frequency and duration; Figure S7A,B) and performed well when a stimulus rate smaller or larger than 2 Hz was used (Figure S7C,F).

Taken together, our network model for deviance detection behaves in a predicted manner in a roving paradigm and when certain populations are removed. Although our network is simplified in many aspects, the network functions robustly when uncertainties that typically appear in biology, such as delays and parameter variabilities, are added.

### 3.3 Rhythmic Activation After Stimulus Cessation Can Be Attained Through STDP‐Driven Entrainment to the Stimulus Rate in a Synfire Chain

The neurons phase‐locked to the stimulus rate were required in our network model for the detection of omissions, but it is unlikely that the auditory pathway constantly has neurons that are phase‐locked to all possible frequencies and multiple phases of these rhythms with a high precision. Here, we show that a network organized as a synfire chain exhibiting STDP in its feedback synapses can be entrained to the rhythmic stimulation in such a way that it faithfully fires rhythmically even after the stimulus cessation.

We simulated a network consisting of a base population that received stimulus encoding auditory stimulation and 70 other neuron populations, each consisting of 50 neurons, that were connected in a feedforward manner (connection probability 0.5) with a 5‐ms transmission delay. Each of the chain populations was weakly connected back to the base population with excitatory synapses that were initially weak but whose strength obeyed a classical STDP rule. The base population received a square‐pulse stimulus every 500 ms (2‐Hz stimulus rate) for 12 s, and each stimulus causes the chain populations to fire in a sequence that lasted approximately 600 ms. We hypothesized that the synapses where the base population fired shortly after the presynaptic neuron were strengthened up to the point that the network is essentially a synfire ring and reverberates in the absence of auditory stimuli, whereas other synapses remain weak or are further weakened.

**FIGURE 4:**
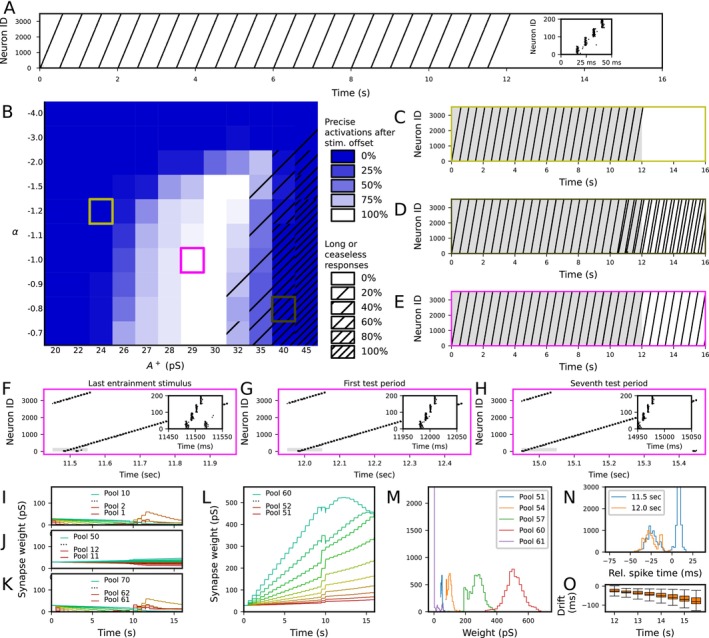
A network organized as a synfire chain with feedback synapses obeying a classical STDP rule can be entrained to 2‐Hz rhythmic stimulus such that it keeps firing in the same rhythm after stimulus cessation. (A) Spike train of the whole network in the absence of STDP, x‐axis representing the time and y‐axis the neuron index. The base population, neurons 1–50, receive rhythmic stimulus every 500 ms, and its activation propagates through the whole synfire chain (neurons 51–3550). Inset: zoomed‐in spike train of the activity in the first four populations during and after the first stimulus. (B) Grid search of the optimal entrainment to the rhythmic stimuli. The x‐axis represents the pre–post addition parameter $A^{+}$, and the y‐axis represents the scaling parameter α ($A^{-}$ = $\alpha A^{+}$). Twenty repetitions of each parameter combination were simulated with different random number seeds. The color of the squares represents the fraction of simulations where spiking activity was observed at the expected time postcessation (12,000 ms + i×500 ms ‐ 60 ms < $t_{\text{spike}}$ ≤ 12,000 ms + i×500 ms + 60 ms, where i=0,1,2,3). Blue represents parameter combinations where none of the 20 simulations produced spiking activity near the times of the expected stimuli postcessation, and white represents parameter combinations where all of them did (a minimum of five spikes per interval was required for a positive hit). The grating of the squares represents the fraction of simulations where spiking activity was observed at a nonexpected time postcessation (12,000 ms + i×500 ms + 60 ms < $t_{\text{spike}}$ ≤ 12,000 ms + i×500 ms + 440 ms, where i=0,1,2,3). The clear squares represent combinations where none of the 20 simulations produced spiking activity (minimum five spikes) between the times of the expected stimuli postcessation, and the fully striped squares represent parameter combinations where all of them did. (C–E) Example spike trains with the parameters indicated in panel B, namely, ($A^{+},\alpha$) = (0.024 nS, −3; C), (0.032 nS, −0.9; D), and (0.028 nS, −3; E). During the first 12 s (gray shaded area), there was a rhythmic 2‐Hz stimulus to the base population. (F–H) Zoomed‐in dynamics on the last stimulus cycle (11.5–12 s; F) and the first (12–12.5 s; G) and seventh (15–15.5 s; H) postcessation cycles from the spike train of (E). The insets show a further zoom‐in on the time of the (actual or expected) stimulus. (I–L) The evolution of the synaptic weights from the 1st to the 10th (I), the 11th to the 50th (J), the 61st to the 70th (K), and the 51st to the 60th (L) populations to the base population. The populations that fired shortly after the base population (such as the 1st, 2nd, 61st, and 62nd pools) were decreased in weight (although in some simulations, they were reincreased after 10 s due to the gradual shortening of the cycle; panels I and K). The 11th to the 50th populations that fired at times that were long apart from the spike times of the base population in both pre–post and post–pre orders remained close to the initial strength (J). By contrast, the 51st to the 60th populations underwent strong increase in synaptic weight due to the entrainment (L). (M) The distribution of synaptic weights at the time of stimulus cessation (t=12 s). The synapses from the 51st, 54th, 57th, and 60th pool represent connections that were strengthened to various degrees due to their activation times while the synapses from the 61st pool, which always activated shortly after the base population, represents a strongly depressed connection. (N) The distribution of activation times of the base population near the last entrainment stimulus (from 20 ms before to 30 ms after 11.5 s; blue) and the first cycle of the expected stimulus postcessation (from 20 ms before to 30 ms after 12.0 s; orange).

To test this hypothesis, we first chose the neuron and synapse parameters that produced a robust synfire chain activity in the absence of STDP (Figure 4A). We then optimized the STDP learning rate parameters $A^{+}$ and $A^{-}$ to permit entrainment to the underlying rhythm. We did this by performing a grid search where $A^{+}$ ranged from 0.02 to 0.045 and $A^{-}$ was chosen as $\alpha A^{+}$, where α ranged from −0.7 to −4.0 (Figure 4B)—we chose the range to be relatively large to allow exploration of different dynamical regimes. We repeated each experiment 20 times with different random number seeds. When $A^{+}$ was small or α strongly negative, the network failed to reach strong enough synaptic weights to continue firing after the entrainment stimulus ceased at T=12 s (Figure 4C). By contrast, when $A^{+}$ was large and $A^{-}$ close to zero, the network became exceedingly excitable and exhibited double activations or even ceaseless spiking responses near the end of the entrainment phase (Figure 4B striped squares; Figure 4D). However, when intermediate values for $A^{+}$ and $A^{-}$ were chosen ($A^{+}=0.029$, $A^{-}=-1.2\times 0.029$), we observed a robust entrainment to the 2‐Hz stimulus rate and the absence of interstimulus activity (Figure 4B white squares and Figure 4E–H). We used these plasticity parameters for the rest of the analyses.

In the networks exhibiting strong entrainment to the 2‐Hz stimulus rate, the 51st to the 60th chain populations were strengthened during the entrainment, while other populations were weakened or little changed (Figure 4I–M). The spiking dynamics of the last stimulus‐accompanied activation cycle (11.5–12.0 s; Figure 4F) were largely similar to those of the first cycle without external stimulus (12.0–12.5 s; Figure 4G), and the sequential activation pattern was preserved even in the seventh cycle without external stimulus (≈15.0–15.5 s; Figure 4H). However, we observed a gradual shortening of the cycle through time (Figure 4H, O). In many simulations, the last stimulus‐evoked response in the base population had a bimodal shape where the newly strengthened synapses made the population fire shortly (≈20 ms) before the arrival of the last stimulus and then again shortly (≈5 ms) after the onset of the last stimulus (Figure 4N; blue). The first non–stimulus‐evoked response had a remarkably similar spike time statistics, apart from the missing reactivation at 5‐ms poststimulus (Figure 4N; orange), consistent with successful entrainment to the 2‐Hz input.

We tested the robustness of our network against changes in stimulus rate. We performed the simulations for stimulus rates 0.75, 1.0, 1.5, 1.75, 2.0, 2.5, 3.0, and 4.0 Hz. Strong entrainment (≥95% of the simulations showed acceptable spike times for at least three cycles after stimulus cessation) was observed for stimulus rates 1.75–3.0 Hz, while stimulus rates 0.75–1.5 Hz did not show activity after stimulus cessation and the stimulus rate 4.0 Hz produced excessive potentiation (Figure S8A). However, if we adapted the length of the synfire chain to be longer for small stimulus rates to span the whole stimulus interval (190 chain populations for 0.75 Hz and 130 chain populations for 1.0–1.5 Hz) and shorter (40 populations) for 4.0 Hz stimulus interval to prevent spanning twice the stimulus interval, we observed successfull entrainment to these stimulus rates too (Figure S8B). A random stimulus protocol (where the stimulus times obeyed a Poisson process with an average 2‐Hz stimulus rate) did not produce any entrainment (Figure S8C).

Taken together, our analysis with rhythmically active point neurons connected with STDP‐exhibiting synapses to the base population suggests that the synapses from the neurons that fire in an optimally timed manner can be significantly strengthened due to entrainment (by, e.g., standard auditory stimuli in the MMN protocol) while other synapses are weakened. In the absence of additional homeostatic mechanisms, our model exhibits a drift toward shortening of the cycle but serves as a proof of concept for a population that becomes phase‐locked to a delta‐band stimulus rate, which is needed for the omission MMN in our modelling framework.

### 3.4 Effects of SCZ Cellular‐Level Phenotypes on Novelty Detection: The Cortical Detection Hypothesis

The network model developed in Section 3.1 is abstract and not directly matched to a specific brain area. Although detection of novelty in auditory stimuli and generation of MMN is likely to be dependent on many brain areas across the whole brain, previous works have highlighted either the contribution of either cortical (Näätänen et al. 2007; Fishman and Steinschneider 2012) or subcortical (Pérez‐González et al. 2005; Malmierca et al. 2009) areas. Since subcortical neurons and circuits are different from their cortical counterparts by both structure and function, the way novelty detection is altered in the schizophrenic brain should be crucially affected by whether the novelty detection takes place in the cortex or subcortical regions. In this section, we explore the effects of SCZ‐associated cellular‐level phenotypes on the predicted novelty detection under the assumption that *all modelled neuron populations are cortical neurons*.

**FIGURE 5:**
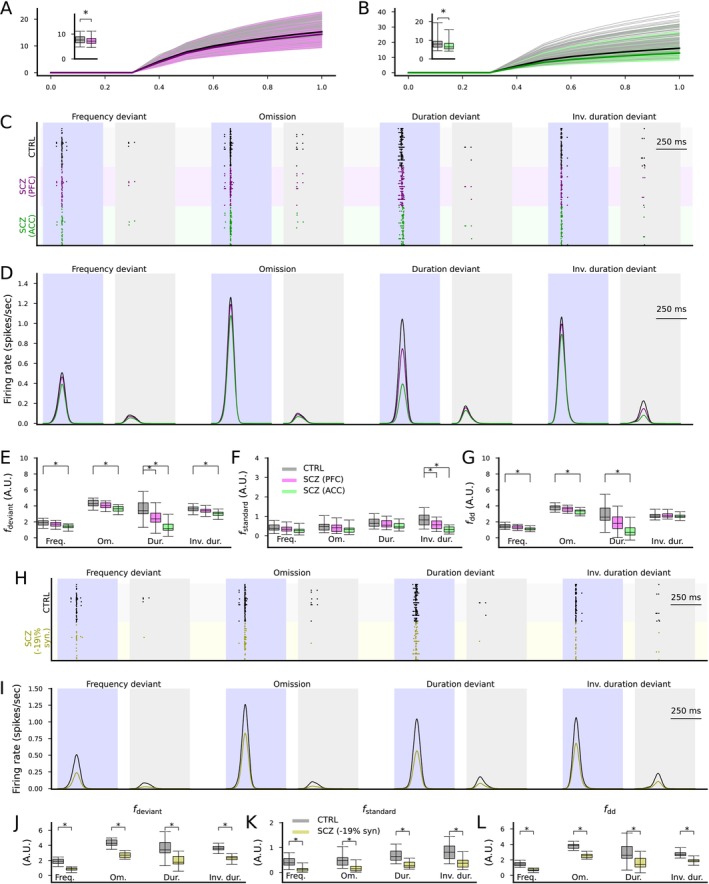
Altered expression of ion channel encoding genes and decreased spine density as measured postmortem in SCZ cortex can impair cortical novelty detection. (A, B) The f‐I curves of the layer V pyramidal cells where ion channel conductances were adjusted based on postmortem RNA expression data in the PFC (A) or ACC (B). Dim gray curves represent control subjects, and dim pink (A) and dim green (B) curves represent SCZ subjects. The thick curves represent the median f‐I curves across the populations (black: control subjects, pink/green: SCZ subjects). Insets: box plot of the AUC across the control (gray) or SCZ (pink/green) subjects. (C) Spike trains of the cortical output population in response to the four protocols (frequency deviant, omission of stimulus, duration deviant, inverse duration deviant) in the CTRL condition (black) and when the membrane capacitance was adapted to SCZ sample simulations according to ion channel expression data from the PFC (purple) or ACC (green). (D) The firing rate curves (smoothed with a Gaussian with an SD of 25 ms) of the spike train data of the three conditions, averaged across the 16 acceptable models. All simulations showed a trend where CTRL response > SCZ PFC response > SCZ ACC response. (E, F) The distribution of the total firing rates in response to each deviant (E) or standard (F) stimulus (from 50 ms before the stimulus onset until 450 ms after the stimulus onset) in the four protocols across the 16 acceptable models. The asterisks show the statistically significant differences between the CTRL and SCZ (PFC or ACC; U‐test, p<0.05/4). (G) The distribution of the novelty detection index calculated from the data of (E) and (F). The asterisks show the statistically significant differences between the CTRL and SCZ (U‐test, p<0.05/4). (H) Spike trains of the cortical output population in response to the four protocols in the CTRL condition (black) and when the excitatory synaptic currents were decreased by 19% to emulate decreased spine density in SCZ (yellow), as reported in auditory cortex (Shelton et al. 2015). (I–L) The experiments of (D)–(G) repeated for CTRL vs SCZ (−19% synaptic conductance).

First, we used the CommonMind data (Hoffman et al. 2019) that included the bulk RNA expression of ion channel encoding genes in the prefrontal cortices (PFC) of 426 subjects (211 SCZ, 215 CTRL) and anterior cingulate cortices (ACC) of 478 subjects (227 SCZ, 251 CTRL). We normalized the expression levels by DESeq2 (Love et al. 2014) and used CIBERSORTx (Steen et al. 2020) to obtain estimates of neuronal expression in these brain regions with the help of a reference single‐cell RNA dataset (Zhang et al. 2016). We focused on genes implicated by GWAS (Trubetskoy et al. 2022) or differential expression that were likely to be expressed in the cortex as done previously (Mäki‐Marttunen et al. 2024).— see Table S1. We calculated subject‐wise factors for each parameter of the Hay model (Hay et al. 2011) affected by these genes. We then performed the subject‐wise single‐cell simulations of neuronal response to a DC of various amplitudes—that is, we estimated the f‐I curves (Figure 5A,B). The AUC were 6.7% and 16.1% smaller in the SCZ compared to CTRL when data from PFC or ACC, respectively, were used (Figure 5A,B). Since firing rate is inversely proportional to the membrane capacitance in the LIAF model (assuming a fixed leak conductance), we implemented these data as our models of SCZ as a change of membrane capacitance parameter: The membrane capacitances of all excitatory neurons (EO, ES, ED, EP, EP2, ESD, EDD populations) were 19.2% or 7.2% (PFC and ACC, respectively) larger in the SCZ case compared to CTRL.

We then performed the network simulations with the four MMN protocols using the altered membrane capacitances (Figure 5C). The simulations showed a decreased response of the output population to both standards and deviants (Figure 5D). The responses to deviants of all types (Figure 5E) and the responses to standards in the inverse duration deviant protocol (Figure 5F) were significantly (p<0.05/4) decreased in amplitude for ACC, and the deviance detection index was significantly decreased in amplitude in the frequency (p=0.00059), omission (p=0.00029), and duration deviant ($p=1.3\cdot 10^{-6}$) protocols but not the inverse duration deviant protocol (p=0.65) for ACC (Figure 5G). For PFC, the response to deviants in the duration deviant protocol (Figure 5E) and the response to standards in the inverse duration deviant protocol (Figure 5F) were significantly (p<0.05/4) decreased in amplitude, but the deviance detection index was not significantly affected (Figure 5G).

We also performed simulations of decreased synaptic density in cortical excitatory neurons (EO, ES, ED, EP, EP2, ESD, EDD populations). Reducing excitatory synaptic conductances by 19% decreased response amplitudes (Figure 5H–K) and significantly reduced the deviance detection index (Figure 5L) across all protocols ($p=6.3\cdot 10^{-8}$ in the frequency deviant and omission, p=0.00044 in the duration deviant, and $p=1.3\cdot 10^{-7}$ in the inverse duration deviant protocol; Figure 5L).

### 3.5 Novelty Detection as a Subcortical Phenomenon: Effects of Cortical SCZ‐Associated Cellular Changes on the Cortical Projection of the Novelty Detection

Although animal studies suggest that auditory novelty detection is stronger in cortical compared to subcortical regions (Lao‐Rodríguez et al. 2024), there is evidence of SSA and/or auditory novelty‐detecting neurons in subcortical regions too, inferior colliculus, and thalamus in particular (Anderson et al. 2009; Pérez‐González et al. 2005; Reches and Gutfreund 2008; Malmierca et al. 2009). Here, we explore the consequences of SCZ‐associated cellular‐level phenotypes on the predicted novelty detection under the assumption that *all modelled neuron populations are subcortical neurons*. Namely, the intrinsic properties and the functionality of our novelty‐detecting network for auditory stimuli could match with a colliculo‐thalamic feedforward network where the output population corresponds to a population in the thalamic MGB, while the other populations correspond to neuronal populations in the IC (see Section 4). However, the effects of SCZ diagnosis on cellular‐level properties (such as expression of ion channels and spine density) as well as circuit abnormalities are reported more often in the cortex than subcortical regions (cf. Lewis and Sweet 2009; Berdenis van Berlekom et al. 2020; Duncan et al. 2025). Likewise, the SCZ‐associated alterations we used in the experiments of Figure 5 were observed in cortical rather than subcortical areas. These data thus only allow us to study how the cortical projection, and possibly postprocessing, of the novelty‐detecting neurons are affected by SCZ although the predicted subcortical dynamics of the novelty detection might occur identically in SCZ compared to CTRL. Therefore, we here introduce an additional, cortex‐like output population that allows us to make model predictions based on these data.

**FIGURE 6:**
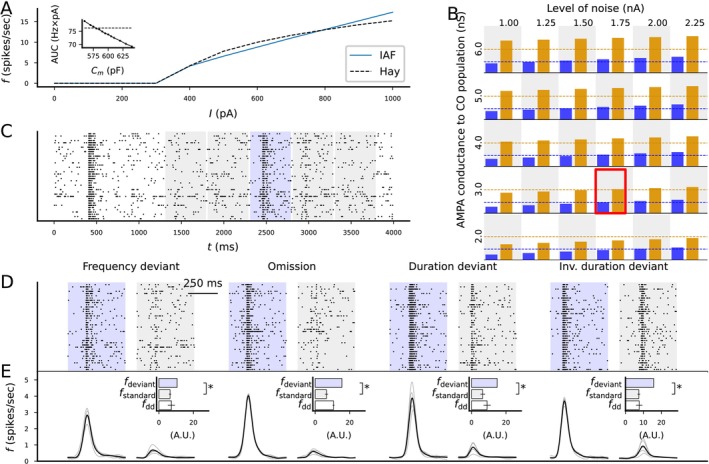
Introduction of a cortical output population and fitting its properties to electrophysiological data from animal oddball experiments.(A) Best fit IAF model to the f‐I curve of the Hay model (Hay et al. 2011) of cortical pyramidal neurons. Inset: AUC (y‐axis) of IAF models with different membrane capacitances (x‐axis). The best fit was obtained by τ=580 ms. (B) Firing rates of the cortical output population in response to standards (blue) and deviants (orange) when different levels of noise and connection strengths from the (subcortical) output population were used. Dashed lines indicate the data recorded from cats (Ulanovsky et al. 2003). Best fit was obtained by a noise level of 1.75 nA and a connection strength of 3 μS. (C) Example spike train of the cortical output population during the frequency deviant protocol. The blue shaded area represents the time interval from which the response to deviants were determined, whereas the gray areas represent the time intervals from which the response to standards was determined. The firing rates from the four responses to standards were averaged. (D) Example spike trains of the cortical output population during the deviant and the first standard after the deviant in all four protocols. (E) Firing rate curves averaged across the 16 models and 20 seeds in response to deviants (left) and standards (right) in all four protocols. The top and middle bars show the mean and SD of the firing rates across 20 random number seeds in response to deviants (blue) and standards (gray). The bottom bar shows the mean and SD of the deviance detection index (white).

We connected the previously described output population (EO) to a cortical output population (CO) of the same size (N=40). We used membrane properties that matched the multicompartmental Hay model of layer V pyramidal cells (Hay et al. 2011), namely, a membrane capacitance of 580 pF and a leak conductance of 4 nS (Figure 6A)—both parameters exhibited a 30% standard deviation within the neuron population centered at these values, similar to other populations (see Section 3.1). To mimic in vivo variability, random membrane currents induced spontaneous fluctuations and spiking. We adjusted the connection strengths from the EO to the CO population and the amplitude of the random membrane currents to match with experimental data on the AP frequency during a response to standards (∼ 8 spikes/s) and oddballs (∼ 20 spikes/s) (Ulanovsky et al. 2003).—the best fit to data was obtained by $g_{AMPA}$ = 3 μS and $I_{noise,SD}$ = 1.75 nA (Figure 6B). We ran the resulting network model and assessed the activity of the cortical output population (Figure 6C) in the different stimulus protocols with all 16 acceptable parameter sets (see Section 3.1). The model provided a strong signal for the novel stimuli in all four stimulus protocols compared to the standards (Figure 6D). In the following, we use the cortical output as the final output affecting the EEG signal and test the effects of different SCZ‐associated cellular‐level alterations on this output.

**FIGURE 7:**
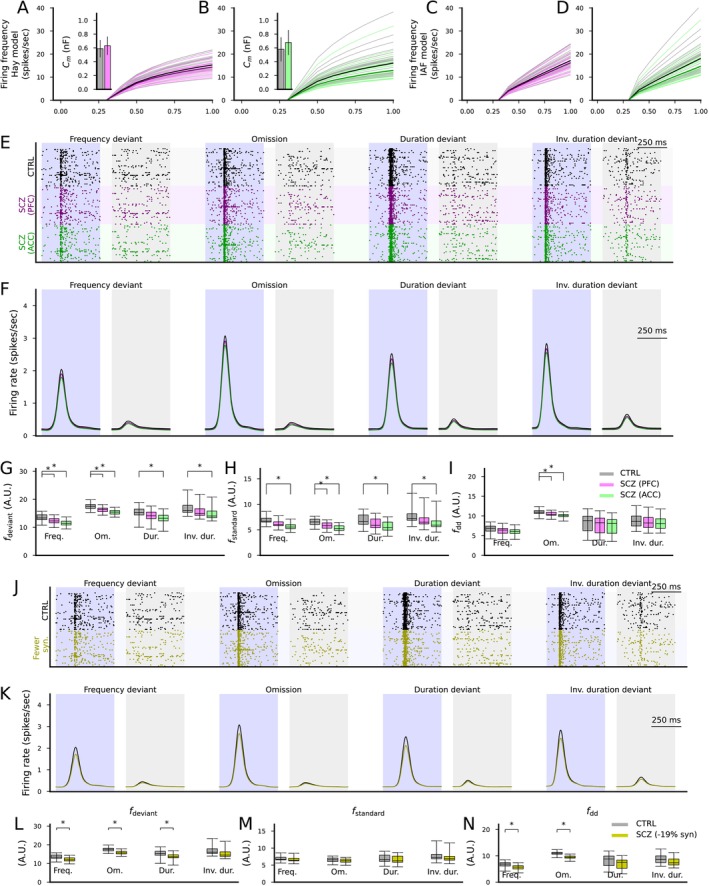
Altered expression of ion channel encoding genes and decreased spine density as measured postmortem in SCZ cortex can impair cortical novelty detection. (A, B) The f‐I curves of the layer V pyramidal cells where ion channel conductances were adjusted based on postmortem RNA expression data in the PFC (A) or ACC (B). Dim gray curves represent control subjects, and dim pink (A) and dim green (B) curves represent SCZ subjects. The thick curves represent the median f‐I curves across the populations (black: control subjects, pink/green: SCZ subjects). Insets: box plot of the AUC across the control (gray) or SCZ (pink/green) subjects. (C, D) The f‐I curves of the best fit IAF models fitted to the data from (A) and (B). (E) Spike trains of the cortical output population in response to the four protocols (frequency deviant, omission of stimulus, duration deviant, and inverse duration deviant) in the CTRL condition (black) and when the membrane capacitance was adapted to SCZ sample simulations according to ion channel expression data from the PFC (purple) or ACC (green). (F) The firing rate curves (smoothed with a Gaussian with an SD of 25 ms) of the spike train data of the three conditions, averaged across the 16 acceptable models. All simulations showed a trend where CTRL response > SCZ PFC response > SCZ ACC response.(G, H) The distribution of the total firing rates in response to each deviant (G) or standard (H) stimulus (from 50 ms before the stimulus onset until 450 ms after the stimulus onset) in the four protocols across the 16 acceptable models. The asterisks show the statistically significant differences between the CTRL and SCZ (PFC or ACC; U‐test, p<0.05/4). (I) The distribution of the novelty detection index calculated from the data of (G) and (H). The asterisks show the statistically significant differences between the CTRL and SCZ (U‐test, p<0.05/4). (J) Spike trains of the cortical output population in response to the four protocols in the CTRL condition (black) and when the excitatory synaptic currents were decreased by 19% to emulate decreased spine density in SCZ (yellow). (K–N) The experiments of (F)–(I) repeated for CTRL vs. SCZ (−19% synaptic conductance).

We next fit the membrane capacitance in the IAF model to each of the subject‐wise adapted simulations of the Hay model (Figure 7A,B) in a similar way as done for the default Hay model in the experiment of Figure 6A. These fits suggested that the membrane capacitance is on average 631 or 678 pF in SCZ according to the PFC or ACC data, respectively (Figure 7A,B insets), compared to the 580 pF fitted for the default Hay model. The IAF model simulations based on these data are illustrated in Figure 7C,D.

We next simulated the novelty‐detecting network with the cortical output population whose membrane capacitance was set to 580 pF (CTRL), 631 pF (SCZ, PFC), or 678 pF (SCZ, ACC). The simulations showed a decreased response of the cortical output population to both standards and deviants (Figure 7E,F). For ACC, responses to both deviants and standards were significantly weaker in SCZ in all protocols, whereas for PFC, the responses to deviants were significantly weaker in the frequency and stimulus omission protocols, and the responses to standards were significantly weaker in the stimulus omission protocol (Figure 7G,H). In both brain areas, the deviance detection index was significantly decreased in the stimulus omission protocol (p=0.00019 in ACC and p=0.012 in PFC) but not other protocols (Figure 7I).

Finally, we simulated the effect of decreased synaptic conductance of the putative thalamocortical synapses on the novelty detection. Similar to the experiments where the synaptic conductances of all excitatory populations were decreased (Figure 5E,F), the decrease in the synaptic conductances of the cortical output population only decreased the response to both standards and deviants (Figure 7J,K). However, the simulations suggested that the decrease in the response to deviants was statistically significant in the frequency deviant, stimulus omission and the duration deviant protocols (Figure 7L) while the decrease in the response to standards was not statistically significant in any protocol (Figure 7M). The deviance detection index was significantly decreased in the frequency deviant and the omission protocol (p=0.0038 and $p=2.2\times 10^{-6}$, respectively) but not in the duration deviant or inverse duration deviant protocols (Figure 7N).

Taken together, our simulations suggest that even if the deviance detection takes place in the subcortical areas, SCZ‐like alterations in the excitatory cortical population receiving the subcortical signals can decrease the novelty detection amplitude. SCZ‐associated ion channel expression differences as measured in the PFC did not significantly affect the deviance detection index, while those measured in the ACC significantly decreased the deviance detection index in the frequency deviant protocol, and the decreased synaptic conductance (to simulate the decrease in numbers of postsynaptic spines in SCZ) decreased the deviance detection index in the frequency deviant and omission protocols.

## 4 Discussion

We developed a spiking network that can detect four types of deviations from standard auditory stimuli: frequency deviants (activating different pitch‐sensitive neurons than standards), stimulus omissions, duration deviants (longer than standards), and inverse duration deviants (shorter than standards). We showed that the network performs robustly in a series of experiments where the stimuli or the neuron properties were mildly altered. We also explored the behavior of the network in the presence of the SCZ‐associated cellular‐level alterations, namely, an altered expression of ion channel encoding genes and an altered density of postsynaptic spines. Our analysis suggests that both a 19% decrease in EPSCs to the excitatory neurons as well as changes in ion channel encoding gene expression that decreases the neuronal excitability can impair the novelty detection but that the former had stronger effects than the latter. This observation was made both when interpreting our novelty detection network to operate cortically (where all excitatory neurons were assumed to be affected by the SCZ‐associated cellular‐level alterations) or mainly subcortically (where only the cortical output population was affected), although the former interpretation led to larger effects altogether.

A key aspect of this study lies in its assumptions and modelling choices. Our model was built in a simplified, yet biophysically meaningful manner, and the modelling choices were primarily made to optimize the model for auditory novel detections. There is thus no evidence of a network in the auditory pathway being strictly organized in the manner we suggested. The suggested neuron populations may therefore represent neurons of multiple neuron types (especially the inhibitory populations) and even neurons at different brain areas rather than a specific anatomical region. However, our modelling choices are not, as far as we are aware, inconsistent with the existing literature. Here, we discuss each of our assumptions and modelling choices in the light of the previous findings and models as well as the alternatives.

1. **Short‐term depressing excitatory synapses between neurons in the auditory pathway.**A central assumption in our model is that novelty detection relies on SSA. Apart from the very upstream cochlear nucleus (Ayala et al. 2013), SSA has been observed in most brain areas in the auditory pathway, including inferior colliculus (IC) (Ayala et al. 2013), superior olivary complex (SOC) (Finlayson and Adam 1997), thalamus (Anderson et al. 2009), and the auditory cortex (Ulanovsky et al. 2003). It is thus natural to hypothesize that neurons encoding for auditory novelty receive direct or indirect inputs from neurons exhibiting SSA. The mechanisms underlying SSA have not been confirmed, but presynaptic short‐term depression has been suggested as a central mechanism for adaptation occurring at time scales larger than 100 ms (Wehr and Zador 2005). We thus built our network to rely on models of presynaptic short‐term depression (Tsodyks and Markram 1997; Wang 1999).
2. **Duration‐sensitive neurons.** In this work, we assumed that the frequency‐tuned neurons are divided into two subgroups: those that only respond to long stimuli and those that respond to both. Such long‐pass firing behaviors have been observed in vivo (Ehrlich et al. 1997; Brand et al. 2000; Sayegh et al. 2011), but there is evidence also of neurons that exclusively respond to short, medium‐duration, or long stimuli (Ehrlich et al. 1997; Aubie et al. 2014). A previous theoretical study suggested that varying the membrane time constant can explain the lag in response to auditory stimulus (Aubie et al. 2012).—we thus implemented the long‐pass duration sensitivity by allowing different membrane time constants (capacitances) to the late‐responding populations (ESD, EDD) that were fitted to optimize the deviance detection. However, if the exclusively duration‐sensitive neurons (as observed in, e.g., Ehrlich et al. 1997; Aubie et al. 2014) are dominant in the auditory pathway, the detection of duration deviants could be implemented in a simpler way where each neuron population responds not only in a frequency‐selective manner but also in a frequency and duration‐selective manner. This would mean that the detection of duration deviants could be conceptually identical to the detection of frequency deviants in our modelling framework: repetitive introduction of tones with a particular frequency and duration depresses the synapses from neurons coding this frequency and duration and a change in either frequency or duration of the tone will activate other neurons whose out‐going synapses are not short‐term depressed.
3. **Existence of neurons that fire phase‐locked to a rate with which standards are presented.** To permit detection of omitted standards, the brain has to know what to predict and, importantly, when to predict. Previous models (e.g., Wacongne et al. 2012; Lieder et al. 2013) have circumvented this problem by assuming a kind of “cache”, that is, a neuron population that saves the memory of the recently income stimulus and replays it with a delay. While there is no direct evidence of such behavior or suggested mechanisms how this memory‐trace firing could emerge, we are not aware of direct evidence for our assumption of delta‐frequency phase‐locked neurons or inputs from such neurons in the auditory pathway either. However, there are findings that indirectly support our assumption. Namely, there are cortical and subcortical neurons that exhibit robust pacemaking activity (reviewed in Bean 2024). For example, the subthalamic neurons showed spontaneous rhythmic firing in the delta‐theta frequency band in vitro (Bevan and Wilson 1999). Likewise, thalamic relay neurons are well‐known for their spontaneous rhythmic generation of bursts of action potentials in a delta‐band frequency (Timofeev et al. 2012). It has also been observed that low‐frequency cortical oscillations can become phase‐locked by introduction of a rhythmic stimulus repeated at a 1.5‐Hz rate (Ten Oever et al. 2017). It is thus natural to hypothesize that standards repeated at a nearby stimulus presentation rate, 2.0 Hz, also make certain population fire at this rate. To argue for the plausibility of our network architecture, we here propose that the phase locking to a rhythmic auditory stimulus may emerge as an entrainment to the stimulus rate through an STDP mechanism in feedback synapses in a network that is otherwise organized as a feedforward synfire chain (Figure 4). However, it remains to be shown that long synfire chains needed for this exist in the brain.
4. **The role of the output population.** The introduction of the output population in all our simulations serves the purpose of showing that it is possible for a single population in the brain to encode auditory novelty of many types in a centralized manner. However, this is not a necessity for the induction of the MMN signal or for shifting the animal's attention toward the cause of the auditory novelty, nor is there evidence that the brain operates this way. The assumption of the existence of such a centralized population is, however, useful for quantifying the network's capacity for novelty detection.

In our SCZ‐applied simulations, we assumed that all neurons were either cortical or subcortical, but, as discussed above, they could be distributed across different cortical and subcortical areas as well. Table S2 shows that optimal detection of some of the four types of deviants may be in partial conflict with that of the other types of deviants, suggesting separate networks for different deviant types, but this issue could also be resolved by using a more complex model where additional model parameters were adjusted. However, one of the brain areas that would match with our modelled novelty‐detecting network both by anatomy and function is the IC. IC receives direct inputs from the upstream auditory areas, i.e., the cochlear nucleus and the superior olivary complex (Adams 1979; Grothe et al. 1994). Approximately 30% of IC neurons are inhibitory, and they connect to both local IC neurons and neurons in other brain areas, including the thalamus (Peruzzi et al. 1997; Oberle et al. 2023). Importantly, IC exhibits strong, rapid inhibition in the form of binaural interaction that is important for sound localization (Sanes et al. 1998).—these same network mechanisms could underlie the inhibition of the phase‐locked population needed for omission detection in our network. IC neurons also display a wide (more than 10‐fold) diversity of excitability‐related properties such as time to spike in response to EPSP onset and threshold stimulus intensity (Tan and Borst 2007), which could give rise to populations responding to long but not short auditory stimulus such as the ES and ED populations described in our model. The phase locking to the 2‐Hz stimulus could possibly be mediated by cortico‐collicular pathway (Lesicko and Geffen 2022), but further research is needed to test this. On the other hand, the requirement for phase locking in the 2‐Hz frequency and the possible requirement of STDP to attain it may fit better to cortical than subcortical circuits.

The existence of neurons in the auditory pathway receiving inputs that are phase‐locked to different phases of the stimulus rate is an assumption that is needed in our framework for the detection of omitted stimuli. In this work, we showed that the timing of these neurons does not need to be perfectly aligned for a successful omission detection (Figure S6D,E) and that the network also tolerates small and medium synaptic delays (Figure 3F,G). We also showed that the phase locking can arise in response to rhythmic auditory stimulation in a feedforward network organized as a synfire chain with feedback connections obeying an STDP rule onto the base population (Figure 4). Although this synfire chain model relies on simplifying assumptions—such as relatively fast propagation (tens of populations within half a second) and high spike‐time precision within a population—it serves as a proof of concept. In future work, the plausibility of this mechanism could be tested in slower and more heterogeneous network models and constrained by experimental data combining wide‐field Ca^2+^ imaging with post hoc structural or molecular microscopy to relate population dynamics to synaptic plasticity markers. If networks operating in this manner exist in the brain and mediate the omission MMN, the deviance detection of omitted stimuli should be weak in the beginning of the MMN experiment, since STDP typically takes many minutes to become fully effective (cf. Bi and Poo 1998). The synfire chains required in this scenario could arise through plasticity rules, as suggested by Zheng and Triesch (2014). In their study, short synfire chains emerged from random connectivity via STDP, and the model also produced short synfire rings (Zheng and Triesch 2014). Alternatively, if populations phase‐locked to many different frequencies and phases natively exist in other parts of the brain, neurons in the auditory pathway could become phase‐locked to one of these population based on the stimulus rate as shown in Luz and Shamir (2016), albeit in different frequency ranges than ours. Note that the learning of rhythmic inputs may also be attained through alternative ways, such as those employed in Socolovsky and Shamir (2021). All these theoretical analyses point to the eligibility of the omission detection mechanisms we propose; however, experimental research is needed to confirm the existence or emergence of phase‐locked inputs to the auditory pathway neurons.

Most of our simulation experiments were carried out using the four MMN protocols that were used in the fitting of the model parameters: short standard to short deviant tone, omission after short standard tones, short standard to long standard tone, and long standard to short standard tone. We also tested that the network successfully detected novelty in all other combinations of the four types of tones that were introduced in a roving paradigm‐like manner and omissions from the other standard sequences (Figure 3A–C). We can expect that the model would function equally well if additional tones were added as alternative stimulus frequencies in addition to the currently implemented “standard” and “deviant” tones. However, the network would not detect more intricate patterns such as the cascade or many‐standards control sequences (Harms et al. 2014) without a more thorough redesign of the network.

SCZ is associated with many cellular‐level phenotypes pertaining to neuron morphology and electrophysiology. In this work, we considered two central alterations affecting pyramidal neuron behavior in the cortex, namely, a 19% loss of synaptic spines (Shelton et al. 2015) and an altered expression of ion channel encoding genes (Hoffman et al. 2019). Beyond these, NMDA receptor hypofunction has long been proposed as a central mechanism underlying MMN deficits, supported by human pharmacological studies where NMDA antagonists such as ketamine reliably reduce MMN amplitudes (Umbricht et al. 2000; Javitt et al. 2008). Dysfunction of GABAergic interneurons, particularly parvalbumin‐positive cells, has also been associated with disrupted excitatory–inhibitory balance and abnormal auditory oscillations (Gonzalez‐Burgos and Lewis 2012; Metzner et al. 2019). These alterations could interact with the excitatory deficits modelled here, potentially compounding their effects. Other reported phenotypes include altered dendritic morphology as well as oligodendrocyte‐ and myelin‐related abnormalities; these changes could further degrade the temporal precision and conduction necessary for omission and duration‐deviant responses (Kalus et al. 2000; Takahashi et al. 2011; Valdés‐Tovar et al. 2022).

At the systems level, empirical findings suggest that not all types of auditory deviants are equally affected in SCZ. Several studies report stronger MMN impairments for duration deviants compared to frequency deviants (Todd et al. 2012; Erickson et al. 2016), a dissociation that resonates with our model's prediction that different deviant types may have different vulnerabilities to cellular‐level alterations. Moreover, the ongoing debate about the relative contributions of cortical versus subcortical structures to MMN generation (Kraus et al. 1992; Grimm and Escera 2012) is directly reflected in our dual interpretations of the network, which yielded converging but distinct predictions about SCZ‐associated impairments.

Our computational analysis is valuable in providing mechanistic in silico predictions against which future observations can be reflected. Pharmacological manipulations that differentially perturb excitatory drive and NMDA receptor function could clarify whether MMN deficits are more strongly driven by excitability loss, synaptic adaptation, or receptor hypofunction. Further validation of our model could be obtained through in vitro slice recordings that test how spine loss and excitability changes shape SSA and omission responses at the circuit level. Finally, patient stratification studies—comparing SCZ subgroups or genetic risk carriers—may reveal whether particular forms of MMN deficits (e.g., omissions versus duration deviants) track with specific cellular phenotypes. Since our model can be tuned to individual gene expression data, it enables personalized predictions of auditory novelty detection deficits and may help disentangle the heterogeneity of SCZ. Given experimental support for the central mechanisms and an identification of the neuron classes behind the proposed populations, our model could then be reused with targeted genetic and structural psychiatric data to better explain the mechanisms of MMN deficits in SCZ, paving way for an improved understanding of the pathophysiology of the disorder.

## Author Contributions

**Ahmed Eissa:** conceptualization (equal); investigation (equal); writing – original draft (equal); writing – review and editing (equal). **Jan Fredrik Kismul:** investigation (equal); writing – review and editing (equal). **Atle Bråthen Pentz:** data curation (equal); writing – review and editing (equal). **Torbjørn Elvsåshagen:** conceptualization (equal); data curation (equal); resources (equal); writing – review and editing (equal). **Christoph Metzner:** conceptualization (equal); writing – review and editing (equal). **Ibrahim Akkouh:** data curation (equal); writing – review and editing (equal). **Srdjan Djurovic:** data curation (equal); resources (equal); writing – review and editing (equal). **Alexey Shadrin:** data curation (equal); writing – review and editing (equal). **Marja‐Leena Linne:** conceptualization (equal); writing – review and editing (equal). **Gaute T. Einevoll:** conceptualization (equal); writing – review and editing (equal). **Ole A. Andreassen:** conceptualization (equal); resources (equal); writing – review and editing (equal). **Tuomo Mäki‐Marttunen:** conceptualization (equal); investigation (equal); project administration (equal); writing – original draft (equal); writing – review and editing (equal).

## Ethics Statement

This research used only previously collected, fully anonymized data from the CommonMind Consortium and did not involve any new human participants or new data collection. Ethical approval from an IRB or equivalent was not required for this secondary analysis, consistent with applicable institutional policies.

## Conflicts of Interest

T.E. received honoraria from Cumulus Neuroscience Ltd. and Sumitomo Pharma America, Inc. O.A.A. is a consultant to HealthLytix and received speaker's honoraria from Lundbeck and Sunovion.

## Supporting information



supplementary.pdf

## Data Availability

The data that support the findings of this study are available from the ModelDB model repository (https://modeldb.science/2019882).
