# Supplementary material for: Computational Modelling of Novelty Detection in the Mismatch Negativity Protocols and Its Impairments in Schizophrenia
Source: Eur J Neurosci. 2026 Mar 20;63(6):e70453. doi: 10.1111/ejn.70453 (PMC13004757; doi:10.1111/ejn.70453)
Supplement: Supplementary file 1 — supplementary.pdf [file EJN-63-0-s001.pdf]

Supplementary figures and tables

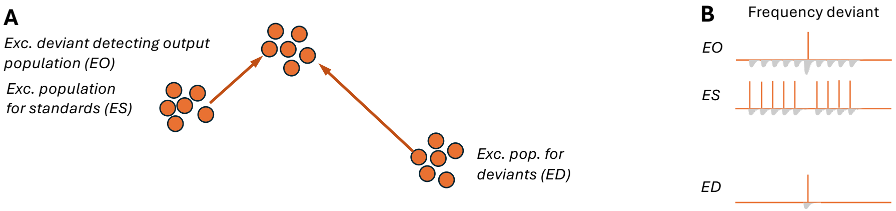

Figure S1: **Illustration of a the hypothesized mechanisms of deviance detection for the frequency deviants and the part of the network required for this.** **A**: Illustration of the part of the network. ES and ED populations project to the EO population with excitatory, short-term depressing synaptic connections. **B**: Illustration of the expected response of the network to a sequence of standard tones, where one of the stimuli is replaced by a deviant tone. The gray EPSCs illustrate the EPSCs received by the neuronal population. The EO population requires **a large EPSC, caused by a first (but not successive) stimulus of the corresponding tone**, to fire.

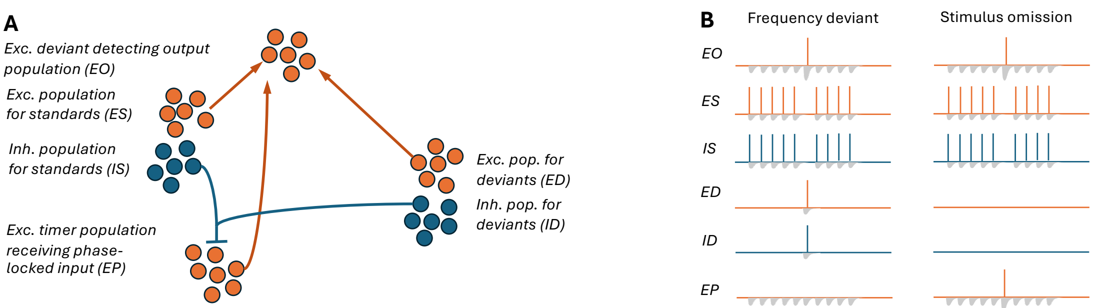

Figure S2: **Illustration of a the hypothesized mechanisms of deviance detection for the frequency deviants and the omitted stimuli and the part of the network required for this.** **A**: Illustration of the part of the network. In addition to the network in Fig. S1, IS and ID populations project to the EP population with inhibitory synaptic connections. The EP population projects to the EO population with excitatory, short-term depressing synaptic connections. **B**: The first column shows the responses to the sequence discussed in Fig. S1, while the second column illustrates the expected response of the network to a sequence of standard tones where one of the stimuli is omitted. The EO population requires either a large EPSC caused by a first stimulus of the corresponding tone **or a large EPSC from the EP population** to fire. The EP is driven by rhythmic inputs arriving at a certain phase of the stimulus rate (2 Hz in this work) but is typically inhibited by auditory stimuli arriving at the same time.

Table S1: Table of ion channel-encoding genes associated with SCZ according to GWAS [Trubetskoy et al., 2022] or post-mortem expression data from the PFC (B) or ACC (C).

**A**<br>Gene	p-value	Affected parameter	Mean effect in PFC	Mean effect in ACC
CACNA1C	1.279e-21	$\bar{g}_{\mathrm{Ca,HVA}}$	+3.73%	+14.87%
HCN1	2.873e-14	$\bar{g}_{\mathrm{Ih}}$	+6.07%	N/A
CACNA1I	1.171e-13	$\bar{g}_{\mathrm{Ca,LVA}}$	+2.86%	+11.20%
KCNB1	2.197e-10	$\bar{g}_{\mathrm{K,p}}$	+7.56%	+10.20%
CACNA1D	3.277e-09	$\bar{g}_{\mathrm{Ca,HVA}}$	+1.65%	+8.29%
KCNQ3	2.128e-06	$\bar{g}_{\mathrm{K,m}}$	+3.68%	+3.89%

**B**<br>Gene	p-value	Affected parameter	Mean effect in PFC
SCN1B	3.238e-08	$\bar{g}_{\mathrm{Na,t}}$	-8.54%
HCN1	0.00031297	$\bar{g}_{\mathrm{Ih}}$	+6.07%

**C**<br>Gene	p-value	Affected parameter	Mean effect in ACC
SCN9A	2.804e-08	$\bar{g}_{\mathrm{Na,t}}$	+17.37%
CACNA1D	3.069e-05	$\bar{g}_{\mathrm{Ca,HVA}}$	+8.29%
CACNA1C	8.836e-05	$\bar{g}_{\mathrm{Ca,HVA}}$	+14.87%
KCND3	9.009e-05	$\bar{g}_{\mathrm{K,p}}$	+5.91%

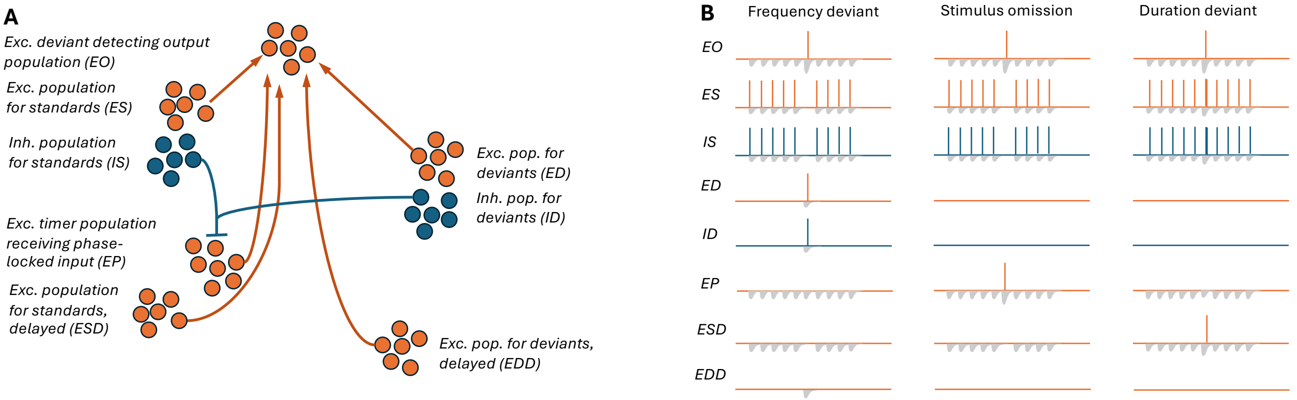

Figure S3: **Illustration of a the hypothesized mechanisms of deviance detection for the frequency and duration deviants and the omitted stimuli and the part of the network required for this.** **A**: Illustration of the part of the network. In addition to the network in Fig. S2, ESD and EDD populations project to the EO population with excitatory, short-term depressing synaptic connections. **B**: The first two columns show the responses to the sequences discussed in Fig. S2, while the third column illustrates the expected response of the network to a sequence of standard tones where one of the stimuli is replaced by a longer stimulus. The EO population requires either a large EPSC caused by a first stimulus of the corresponding tone, a large EPSC from the EP population, **or an EPSC from both ES/ED and ESD/EDD** to fire. The ESD and EDD populations are activated by the same auditory stimuli as ES and ED, but they require a longer presentation of the stimulus (i.e., a longer integration of the corresponding inputs) to fire.

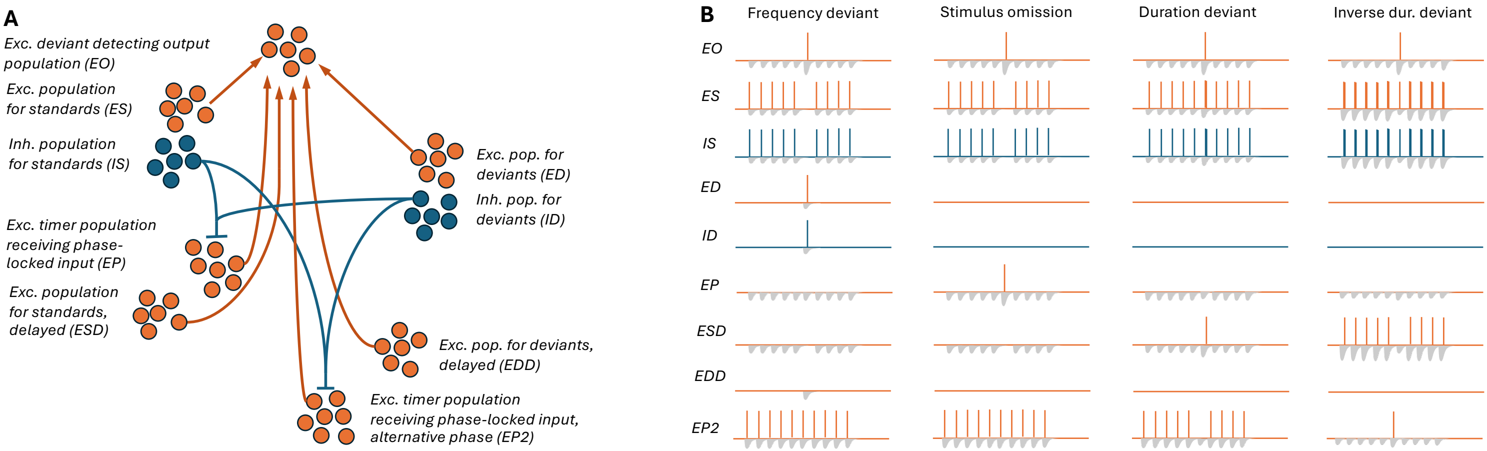

Figure S4: **Illustration of a the hypothesized mechanisms of deviance detection for all deviants.** **A**: Illustration of the part of the network. In addition to the network in Fig. S3, IS and ID populations project to the EP2 population with inhibitory synaptic connections. Similar to the EP population, the EP2 population projects to the EO population with excitatory, short-term depressing synaptic connections. **B**: The first three columns show the responses to the sequences discussed in Fig. S3, while the fourth column illustrates the expected response of the network to a sequence of long standard tones where one of the stimuli is replaced by a shorter stimulus. The EO population requires either a large EPSC caused by a first stimulus of the corresponding tone, a large EPSC from the EP **or EP2** population, an EPSC from both ES/ED and ESD/EDD to fire. The EP2 is driven by rhythmic inputs arriving at a certain phase (prior to the preferred phase of the EP population) of the stimulus rate but is typically inhibited by long, auditory stimuli arriving at the same time. The short stimulus deviant allows EP2 to fire, causing EO to fire as well. The EP2 population fires typically in the other protocols (first to third column), but the inputs caused by these activations become short-term depressed and thus cause no firing of EO population.

Table S2: Parameters and their ranges used in the grid search (A), the acceptable parameter sets obtained (B), and the means and SDs of the set of acceptable parameters for each protocol separately and together (C). The default model parameters are printed in bold.

A

P1: Stimulus amplitude (ES, IS, ED, ID, ESD, EDD)	120	125	130	140	150	(pA)	
P2: Synaptic conductance from ES and ED to EO	12.5	15.0	17.5	20.0	22.5	(nS)	
P3: Synaptic conductance from EP and EP2 to EO	25.0	30.0	35.0	40.0	45.0	(nS)	
P4: Synaptic conductance from ESD and EDD to EO	80.0	90.0	100.0	110.0	120.0	130.0	140.0 (nS)
P5: NMDA to AMPA ratio	0.5	0.333					
P6: Synaptic conductance from IS and ID to EP and EP2	10.0	15.0	20.0	25.0	30.0	35.0	(nS)
P7: Strength of depression p_v	0.9	0.95					
P8: Average membrane capacitance of ESD and EDD	200	250	(pF)				

Other model parameters:
Stim. amplitude of EP and EP2: 20 pA smaller than the stimulus amplitude of ES, IS, ED, ID, ESD, EDD (P1).
Time constant of recovery from short-term depression: 1000 ms.
Average membrane capacitances (C_m) of ES, IS, ED, ID, EP, and EP2: 10 pF.
SD of membrane capacitance (C_m) within a population (all populations): 0.3×mean(C_m)

B

	P1	P2	P3	P4	P5	P6	P7	P8
**Parameter set 1**	**150**	**17.5**	**30.0**	**80.0**	**0.333**	**35.0**	**0.95**	**250.0**
Parameter set 2	150	17.5	30.0	80.0	0.333	35.0	0.9	250.0
Parameter set 3	150	17.5	30.0	80.0	0.333	30.0	0.95	250.0
Parameter set 4	150	17.5	30.0	80.0	0.333	30.0	0.9	250.0
Parameter set 5	150	17.5	30.0	80.0	0.333	25.0	0.95	250.0
Parameter set 6	150	17.5	30.0	80.0	0.333	25.0	0.9	250.0
Parameter set 7	150	17.5	30.0	80.0	0.333	20.0	0.9	250.0
Parameter set 8	150	17.5	30.0	80.0	0.333	15.0	0.95	250.0
Parameter set 9	150	17.5	30.0	80.0	0.333	15.0	0.9	250.0
Parameter set 10	140	17.5	30.0	80.0	0.333	35.0	0.9	250.0
Parameter set 11	140	17.5	30.0	80.0	0.333	30.0	0.9	250.0
Parameter set 12	140	17.5	30.0	80.0	0.333	10.0	0.9	250.0
Parameter set 13	125	17.5	40.0	140.0	0.333	25.0	0.9	250.0
Parameter set 14	125	17.5	40.0	140.0	0.333	10.0	0.9	250.0
Parameter set 15	125	17.5	40.0	100.0	0.333	25.0	0.9	200.0
Parameter set 16	125	17.5	40.0	100.0	0.333	10.0	0.9	200.0

C

Protocol	#accepted parameters	P1	P2	P3	P4	P5	P6	P7	P8
Freq. deviant	7249	129±9	14.8±2.3	35.8±5.3	109±20	0.38±0.08	23.0±8.6	0.93±0.02	226±25
Omission	10118	130±10	20.3±2.0	31.2±5.9	110±20	0.41±0.08	22.6±8.6	0.93±0.02	225±25
Dur. deviant	15022	131±10	17.4±3.5	29.2±4.4	111±20	0.40±0.08	22.5±8.5	0.93±0.02	220±24
Inv. dur. deviant	7199	126±7	17.4±3.5	41.3±3.9	101±19	0.41±0.08	22.5±8.5	0.93±0.02	238±21
All protocols:	16	142±10	17.5±0.0	32.5±4.3	90±20	0.33±0.00	23.4±8.8	0.91±0.02	244±17

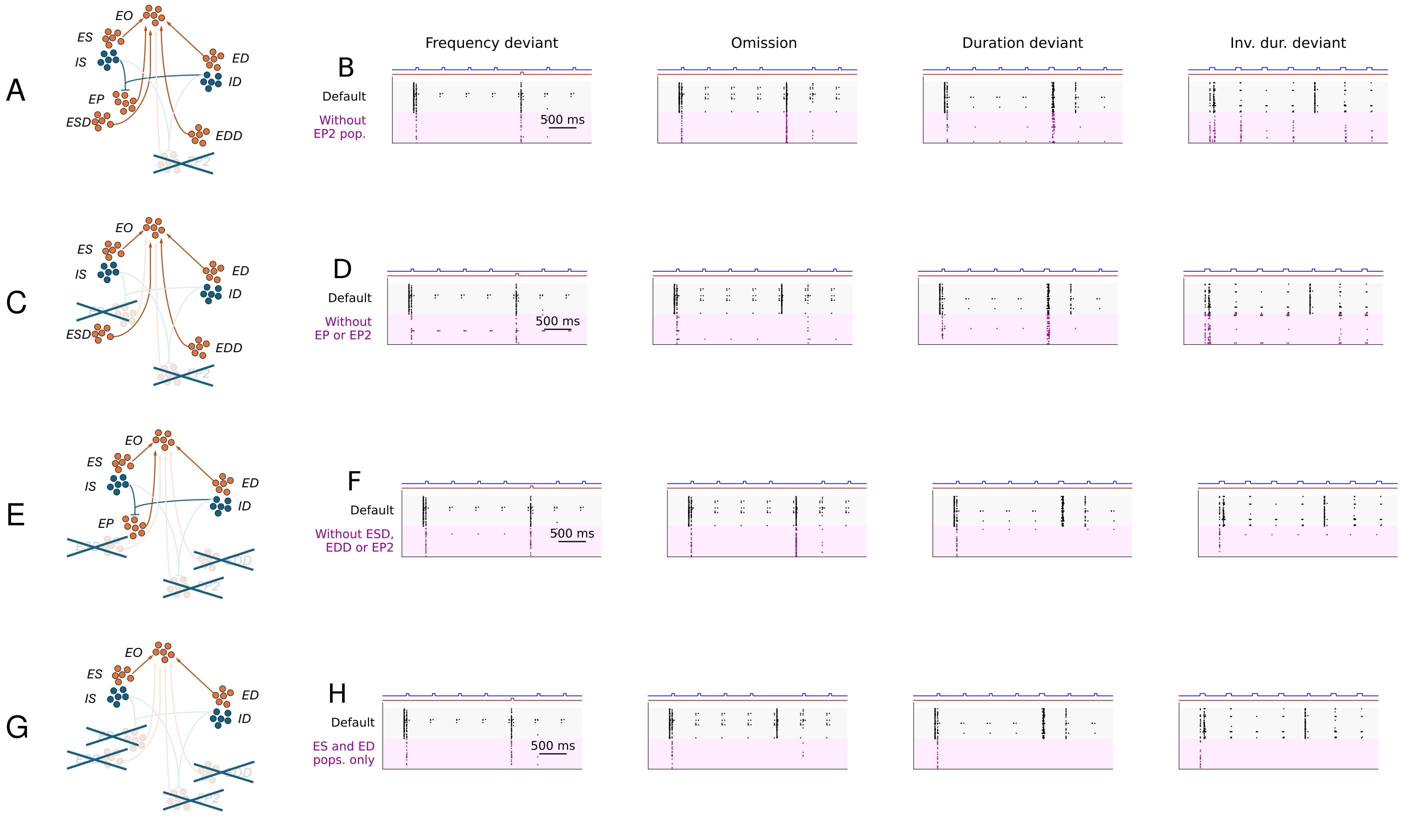

Figure S5: **The removal of subpopulations leads to loss of deviance detection in different protocols. A,C,E,G**: Illustration of the network and the removed populations (crossed out). A: The population EP2 removed. C: The EP and EP2 populations removed. E: The ESD, EDD, and EP2 populations removed. G: The ESD, EDD, EP and EP2 populations removed (this is equal to also IS and ID being removed since they no more interact with any remaining population). **B,D,F,H**: The population spike train of the output population in the default (black) network and in the network where the population(s) indicated in panels A, C, E, and G, respectively, were removed (magenta). The spike trains indicate that the deviance detection was lost in the inverse duration deviant (B), the omission and the inverse duration deviant (D), the duration and inverse duration deviant (F), or the omission, duration and inverse duration deviant (H) protocols.

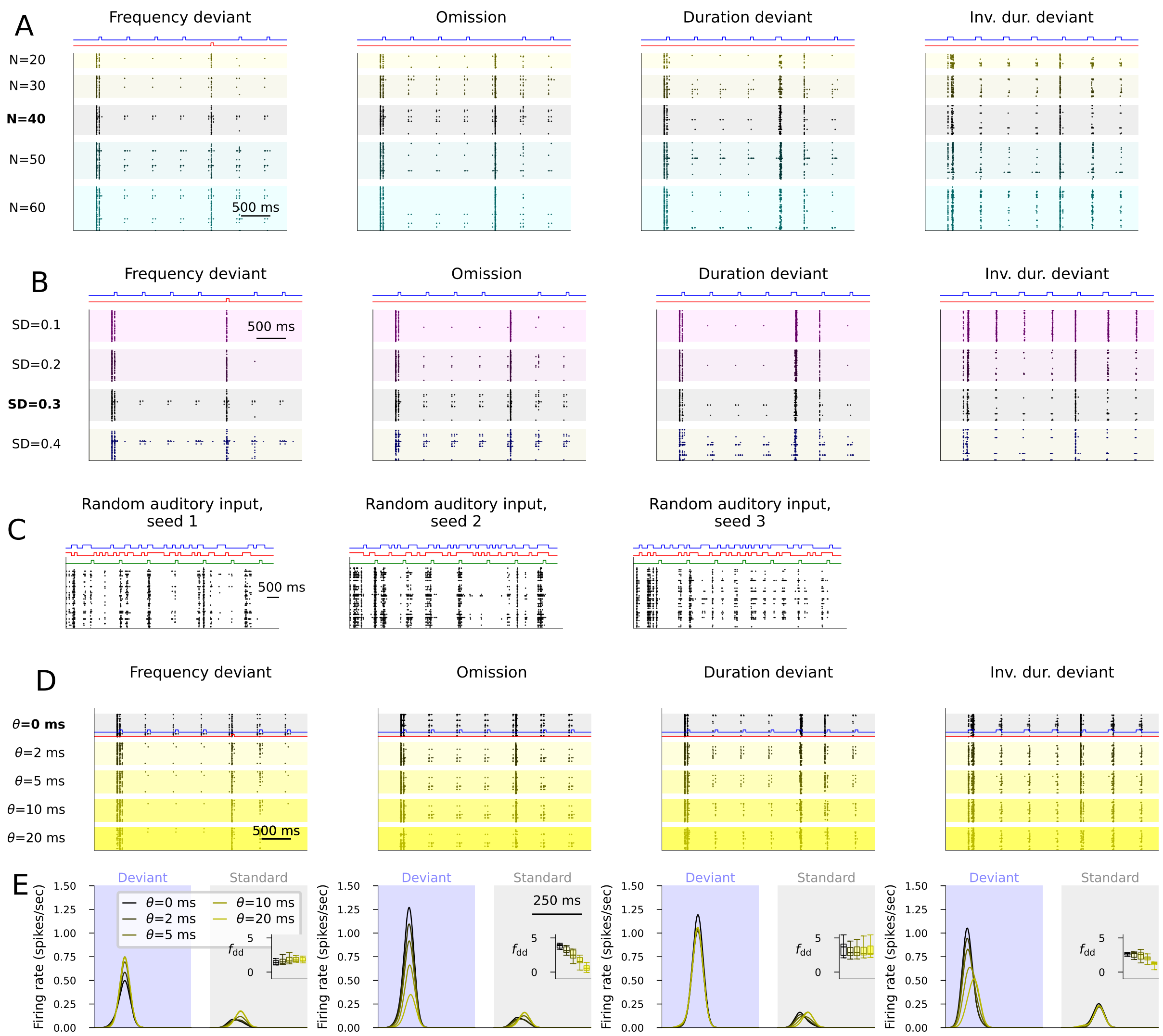

Figure S6: **The deviance detection network is robust against changes in network size and intrinsic variability as well as against small jitter in the phase of the phase-locked populations.** **A**: The population spike trains of the output population when different population sizes were used. The default parameter (N=40 neurons per population) is shaded in gray. In each experiment with an altered network size, all synaptic conductances were compensated by an inverse factor (i.e., when network size was changed from 40 to 60, synaptic conductances were divided by 1.5 from their default value). **B**: The population spike trains of the output population when different intrinsic variabilities of the membrane capacitance (C_m) were used. The default parameter (SD(τ)=0.3× mean(τ)) is shaded in gray. Slightly altered variabilities (SD=0.2, 0.4) resulted in successful deviance detection, but a network where all neurons within a population had exactly the same membrane capacitance (SD=0) resulted in a compromised detection of inverse duration deviants. **C**: An experiment where the auditory inputs are random does not show artifacts caused by the phase-locked populations. The three panels show the results from experiments performed with three different random number seeds. **D**: Experiments where the phase-locked neurons had a jitter of up to 0 (default), 2, 5, 10, or 20 ms around their expected time of firing. **E**: The experiments of (D) repeated for all 16 models and 10 repetitions with different random number seeds. The insets show the deviance detection indices for the jitter of 0 (left), 2, 5, 10, and 20 ms (right). The detection of omissions is compromised for medium (10 ms) and large (20 ms) jitter, and the detection of inverse duration deviants is impaired for large (20 ms) jitter, while the detection of frequency and duration deviants is unaffected by the jitter.

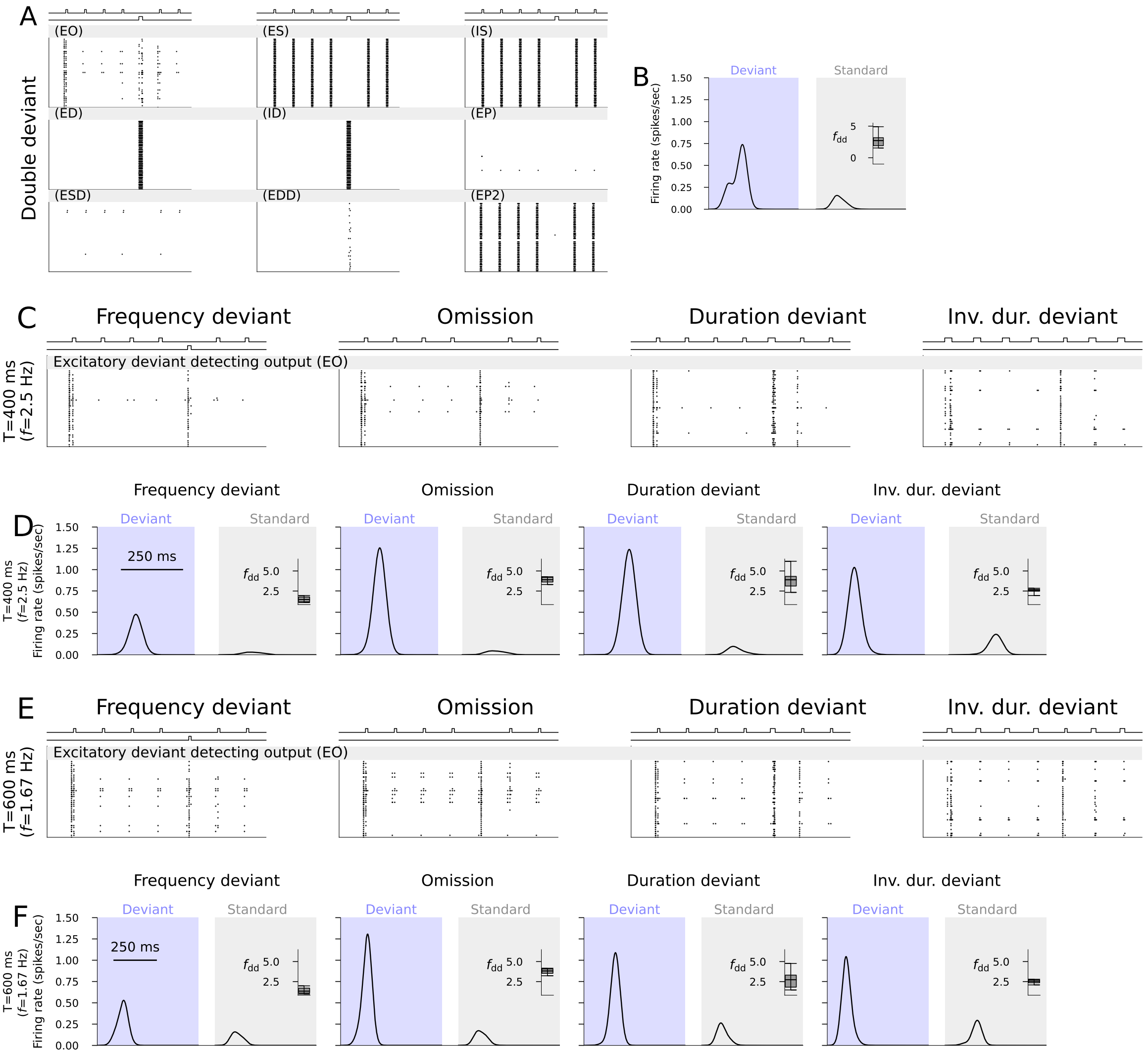

Figure S7: **The deviance detection network functions robustly when a double deviant is used or when a 2.5 or 1.67 Hz stimulus rate is used. A**: The activation of the nine populations in response to the double deviant (long deviant tone preceded by a sequence of short standard tones). **B**: The firing rate curve in response to the double deviant, compared to that elicited by the standard. See Fig. 3E for details. **C**: Population spike raster plots of the nine subpopulations in the double-deviant (longer duration and different frequency) protocol according to one of the 16 acceptable models. **D**: Average firing rate curves induced by the double deviant (blue shading) and those induced by the previous tone (gray shading) in the double-deviant protocol, see Fig. 3E for details. The insets show the box plots of the deviance detection indices. **E**: Population spike raster plots of the output population in response to the four MMN protocols when a 2.5 Hz (inter-stimulus interval 400 ms) stimulus rate was used, according to one of the 16 acceptable models. **F**: Average firing rate curves induced by the double deviant (blue shading) and those induced by the previous tone (gray shading) in the double-deviant protocol, see Fig. 3E for details.

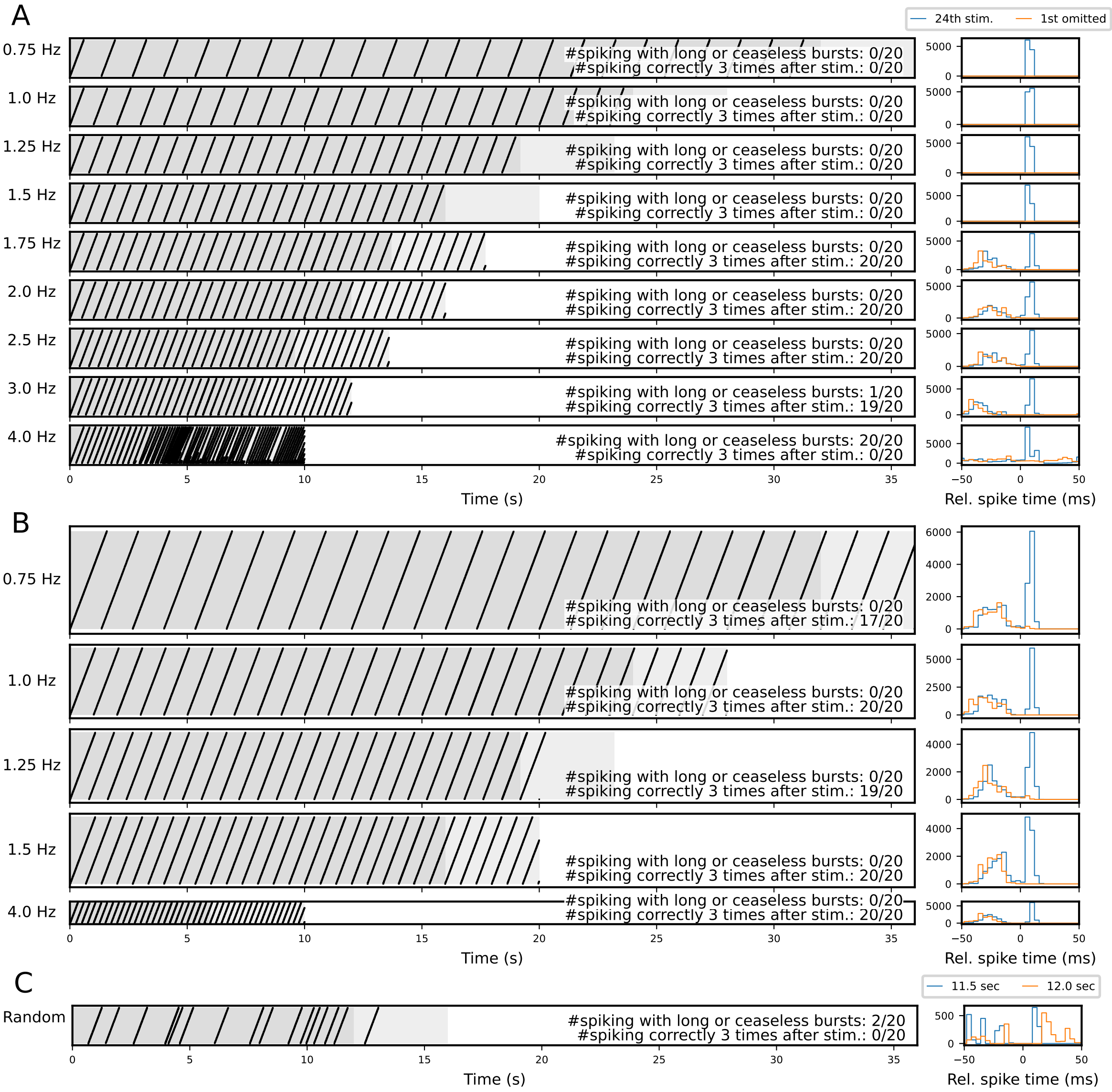

Figure S8: **The entrainment of the synfire chain network with feedback synapses that can be strengthened through the STDP mechanism is successful for nearby stimulus rates.** **A**: Left: An example population spike train from simulations where different stimulus rates (0.75–4 Hz) were used in the default-size synfire chain. The simulations consisted of 24 cycles of stimulated activity (12 seconds for 2 Hz, 32 seconds for 0.75 Hz, and 6 seconds for 4 Hz) and a fixed 4-second test period. A single simulation is shown for each stimulus rate. In each panel, the statistics of successful entrainment (at least three appropriately timed bursts, see Methods and Fig. 4B) and long population bursts across 20 simulations with different random seeds are printed. Right: the distribution of the spike times near the last stimulus and the first expected (omitted) stimulus. See Fig. 4N for details. **B**: Left: An example population spike train from simulations where different stimulus rates (0.75, 1.0, 1.25, 1.5, and 4.0 Hz) were used in longer (190 chain populations for 0.75 Hz, and 130 chain populations for 1.0, 1.25, and 1.5 Hz) or shorter (40 chain populations for 4.0 Hz) synfire chains. Right: the distribution of the spike times across 20 simulations. See panel (A). **C**: Left: An example population spike train from simulations with a default-size synfire chain where the stimulus is a Poisson process with an average 2-Hz rate for the first 12 seconds. Right: the distribution of the spike times across 20 simulations. See panel (A).
